# Coilin mediates m^6^A RNA methylation through phosphorylation of METTL3

**DOI:** 10.1242/bio.060116

**Published:** 2023-12-05

**Authors:** Douglas M. McLaurin, Sara K. Tucker, Michael D. Hebert

**Affiliations:** Department of Cell and Molecular Biology, The University of Mississippi Medical Center, Jackson, MS 39216-4505, USA

**Keywords:** Cajal body, Coilin, METTL3, Methylation, MicroRNA

## Abstract

MicroRNAs (miRNAs) are a class of noncoding RNAs that regulate gene expression. An important step in miRNA biogenesis occurs when primary miRNAs are bound and cleaved by the microprocessor to generate precursor miRNAs. Regulation at this step is essential and one such regulator includes m^6^A RNA methylation, an RNA modification found on primary miRNAs that is installed by METTL3 and bound by hnRNPA2B1. Our lab has recently discovered that the Cajal body marker protein coilin also participates in miRNA biogenesis and hypothesized that coilin may be influencing miRNA biogenesis through m^6^A RNA methylation. Here we report that coilin suppression reduces m^6^A on primary Let7a and miR-21. We also found that coilin suppression reduced the protein expression of hnRNPA2B1 and METTL3. We observed an interaction between coilin and ectopically expressed METTL3 and found that coilin suppression reduced the nucleoplasmic portion of METTL3 and blunted ectopic METTL3 phosphorylation. Finally, coilin suppression disrupted the greater METTL3 complex with cofactors METTL14 and WTAP. Collectively, our work has uncovered a role for coilin in mediating m^6^A RNA methylation and provides an avenue by which coilin participates in miRNA biogenesis.

## Teaser

**Summary:** Our work highlights a novel role for the Cajal body marker protein, coilin, in m6A RNA methylation by which it influences the expression of METTL3 and participates in microRNA biogenesis.

## INTRODUCTION

MicroRNAs (miRNAs) are a class of noncoding RNAs that regulate gene expression at the RNA level and play a pivotal role in a wide array of biological processes (Saliminejad et al., 2019; Taganov et al., 2007; Small and Olson, 2011; Ma et al., 2011). The biogenesis of miRNAs can be captured into three phases: formation of the primary transcript (pri-miRNA), formation of the precursor transcript (pre-miRNA), and formation of the mature transcript. An important step in the biogenesis pathway occurs at the second phase when the pri-miRNA is bound and cleaved to produce pre-miRNA (Saliminejad et al., 2019). This activity is catalyzed by the microprocessor complex, which consists primarily of Drosha and DGCR8 (Han et al., 2004; Lee et al., 2003). Blockage at this step results in suppression of the mature miRNA coupled with an accumulation of the pri-miRNA (Han et al., 2004; Lee et al., 2003).

The m^6^A RNA methylation of pri-miRNAs has recently been uncovered as a novel regulator of miRNA biogenesis (Alarcón et al., 2015a,b). The regulatory network controlling m^6^A RNA methylation involves three groups of proteins: *writers*, which install m^6^A modifications; *erasers*, which remove m^6^A modifications; and *readers*, which bind to m^6^A modified RNAs and guide them along their maturation pathway (He et al., 2019). It has been discovered that the *writer* methyltransferase-like 3 (METTL3) and the *reader* hnRNPA2B1 modifies and binds pri-miRNAs, respectively (Alarcón et al., 2015a,b). As such, these proteins act as positive regulators of miRNA biogenesis, and their suppression decreases the production of mature miRNAs and leads to an accumulation of pri-miRNAs (Alarcón et al., 2015a,b).

Our lab has also uncovered a role for the Cajal body (CB) marker protein coilin as a positive regulator of miRNA biogenesis (Logan et al., 2021, 2022, 2020; Lett et al., 2021). Canonically, coilin function has been largely associated with its CB marker role, wherein coilin maintains CB assembly (Bohmann et al., 1995; Hebert and Matera, 2000; Hebert et al., 2002) and spliceosome efficiency (Bellini and Gall, 1998; Sleeman et al., 2001; Tucker et al., 2001). However, many noncanonical functions have been described for coilin. Some of these functions include a role in the DNA damage response (Velma et al., 2010, 2012; Bartova et al., 2014), neuronal differentiation (Krejci et al., 2017; Forthmann et al., 2013), and the viral response (Shaw et al., 2014; Shaw et al., 2019; Makhotenko et al., 2019). In wanting to understand how coilin might participate in miRNA biogenesis, our lab has investigated coilin's interaction with the microprocessor complex. To summarize, we have found that coilin suppression reduces DGCR8 phosphorylation and SUMOylation, resulting in decreased protein stability and blunting of microprocessor activity (Lett et al., 2021, 2023).

Here, we report that in addition to regulating DGCR8 stability, coilin mediates m^6^A RNA methylation. We found that coilin suppression results in a reduction of m^6^A RNA methylation of primary Let7a and miR-21 miRNAs. We also found that coilin suppression reduced the RNA and protein expression of hnRNPA2B1, but induced the RNA expression while reducing the protein expression of METTL3. We observed an interaction between coilin and ectopic METTL3 and found that coilin suppression reduced METTL3 within the nucleoplasm and depressed phosphorylation of ectopic METTL3 at Serine 43. Finally, coilin suppression disrupted the METTL3-METTL14-WTAP complex. Collectively, our work has uncovered a role for coilin in mediating m^6^A RNA methylation and provides an additional pathway in which coilin participates in overall miRNA biogenesis.

## RESULTS AND DISCUSSION

### Coilin knockdown reduces m^6^A RNA methylation of primary-Let7a and primary-miR-21

**Fig. 1.**
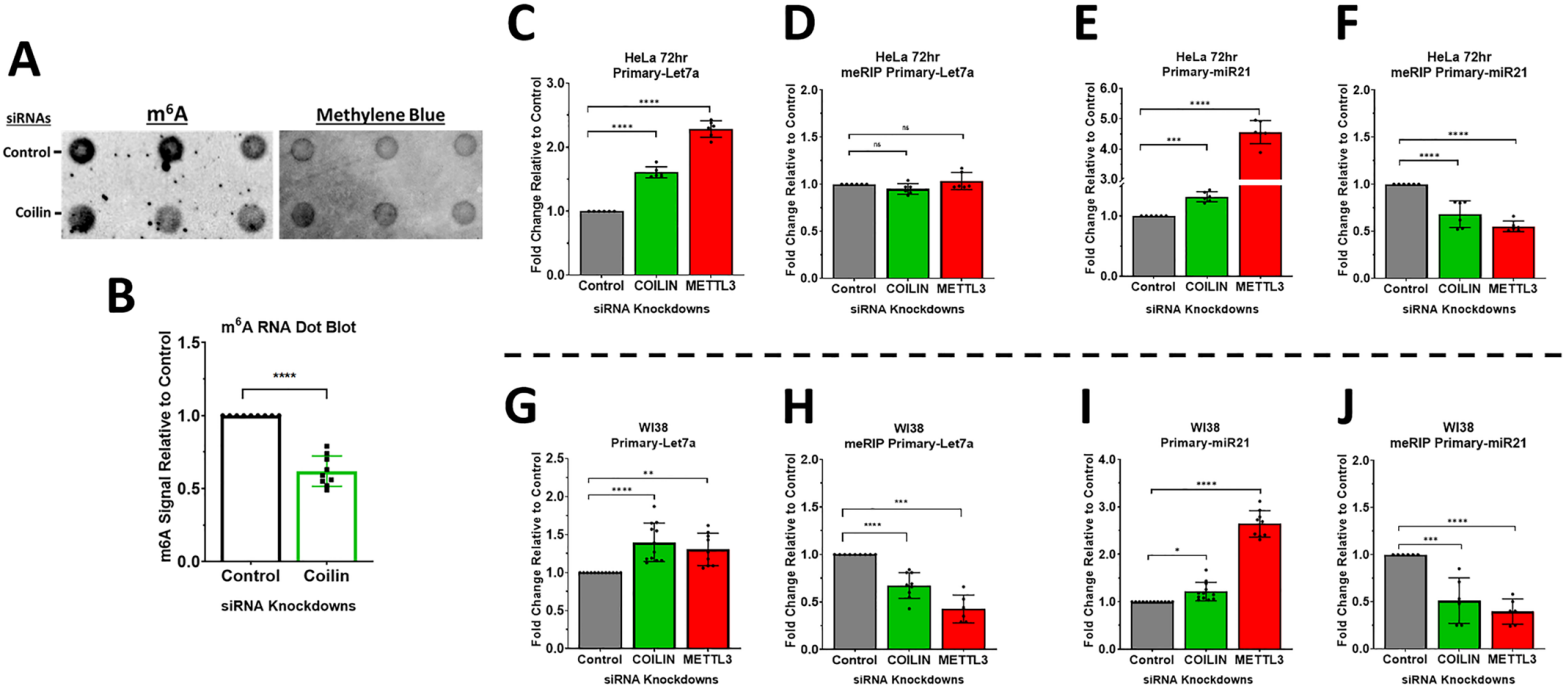
**Coilin suppression reduces m^6^A modification of primary Let7a and miR-21 microRNAs.** (A) HeLa cells were transfected with siRNAs for 96 h. RNA was collected and 500 ng was dotted onto a positively charged nylon membrane and probed with anti m^6^A. (B) The signal intensities of m^6^A and Methylene Blue were assessed and the m^6^A/mb ratio was normalized to control with the control set to 1. Data represent three biological replicates with three technical repeats (*N*=9). siRNAs were transfected into HeLa cells (C-F) or WI38 cells (G-J) for 72 h. Total RNA was collected and 5 μg was used for meRIP using anti m^6^A followed by a second RNA extraction. RNAs were subject to qRT-PCR. Data represent three biological replicates with two to three technical repeats (*N*=6-9). Error bars represent s.d. and black points represent individual data points. *P<0.05, ***P<0.001, ****P<0.0001, ns=not significant.

To determine if coilin has an effect on m^6^A RNA methylation, we examined m^6^A via dot blot assay using total RNA extracted from HeLa cells (Fig. 1A). After the initial m^6^A probing, we then stained the membrane with methylene blue and used this to calculate the 40% reduction in m^6^A RNA methylation upon coilin knockdown (KD) (Fig. 1B). While more quantitative analyses such as LC/MS-MS or meRIP will be needed for validation and targeted assessments on the global cell level, this result suggests that coilin may play a role in the m^6^A RNA methylation of numerous RNA types within the cell.

Because we had a specific interest in the modification of primary miRNAs, we next turned to m^6^A RNA immunoprecipitation (meRIP) coupled with qRT-PCR. For the meRIP assay, our qRT-PCR targets were pri-Let7a and pri-miR-21. Pri-Let7a is a miRNA whose biogenesis our lab has found to be regulated by coilin (Logan et al., 2020; Lett et al., 2021) and pri-miR-21 is a novel miRNA whose coilin-mediated biogenesis is being investigated for the first time. Both miRNAs have been previously described as being regulated by m^6^A modification (Konno et al., 2019; Ye et al., 2022). Additionally, meRIP assays were conducted in both HeLa cells, a cancer cell line, and WI38 cells, a primary cell line. CBs are not observed in all cell types, and even when CBs are present, ∼70% of coilin can still be found within the nucleoplasm (Sawyer et al., 2017; Lam et al., 2002; Young et al., 2001). This suggests that coilin has functions independent of the CB. Thus, we were interested in determining whether differences in the nuclear organization of coilin might influence its effect on m^6^A. Therefore, examining coilin's effect on m^6^A in the WI38 primary cell line in which few CBs form (Spector et al., 1992) would allow for a contrast against the HeLa cancer cell line in which CBs are abundant (Spector et al., 1992). For additional controls, we included the KD of METTL3 as a positive control for downregulation of m^6^A.

In HeLa cells, coilin and METTL3 were KD for 72 h and RNA was collected for qRT-PCR of total RNA and RNA collected after meRIP. In total RNA, we observed that both coilin and METTL3 KD for 72 h upregulated the expression of pri-Let7a and pri-miR-21 (Fig. 1C,E). In RNA collected after meRIP, we observed no change in the recovery of pri-Let7a (Fig. 1D) and a decrease in the recovery of pri-miR-21 (Fig. 1F) upon coilin and METTL3 KD. The results for the meRIP of pri-Let7a were unexpected, especially when compared to that of pri-miR-21. Curious about whether the KD period would have an impact on the m^6^A of pri-Let7a, we extended the KD period to 96 h and again collected total RNA and RNA after meRIP. In total RNA, we again observed an induction of pri-Let7a upon coilin and METTL3 KD (Fig. S1A). In RNA collected after meRIP, we continued to observe no change upon coilin KD, but we did observe a significant decrease upon METTL3 KD at 96 h (Fig. S1B).

In WI38 cells, coilin and METTL3 were also KD for 72 h and RNA was collected for qRT-PCR of total RNA and RNA collected after meRIP. In total RNA, we observed an induction of both pri-miRNAs upon coilin or METTL3 KD (Fig. 1G,I). In RNA collected after meRIP, we observed a decrease in the recovery of both pri-miRNAs as a result of suppressed m^6^A RNA methylation (Fig. 1H,J). We have confirmed that the siRNA targets in these data achieve an appropriate KD (Fig. S2).

These results are in line with previous observations of METTL3's role in miRNA biogenesis wherein METTL3 suppression results in an upregulation of pri-miRNA levels with a corresponding decrease in m^6^A modification of the pri-miRNA and decreased production of the mature miRNA (Alarcón et al., 2015a,b). Similar to METTL3, coilin suppression has also been found to result in the upregulation of the pri-miRNA transcript with a corresponding decrease of the mature transcript (Logan et al., 2021, 2022, 2020; Lett et al., 2021). However, the observation that the recovery of m^6^A-modified pri-Let7a and pri-miR-21 is depressed upon coilin KD is a new and quite interesting discovery and suggests that the m^6^A pathway could be a mode in which coilin participates in miRNA biogenesis.

### Coilin knockdown dysregulates the expression of METTL3 and hnRNPA2B1

**Fig. 2.**
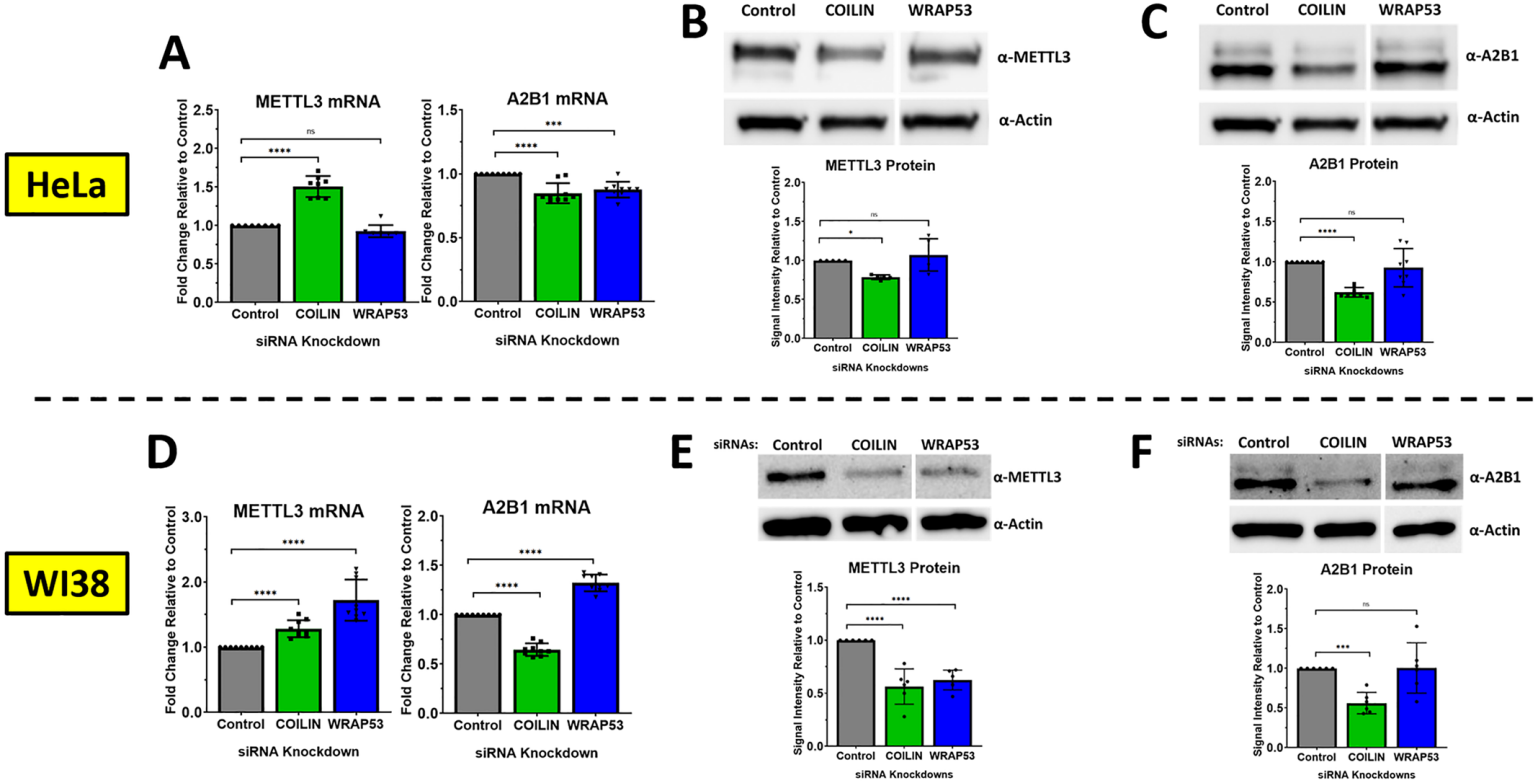
**Coilin suppression dysregulates expression of METTL3 and hnRNPA2B1.** siRNAs were transfected into cells for 72 h. RNA or protein extracts were collected and subject to qRT-PCR or Western blot, respectively. For qRT-PCR (A,D), targets included GAPDH, METTL3, and hnRNPA2B1. For Western blot (B-C, E-F), signal intensities were determined for the targets (METTL3, hnRNPA2B1) and Beta-Actin. A target/actin ratio was calculated and normalized to control with control set to 1. All qRT-PCR data represent three biological replicates with two to three technical repeats (*N*=8-9). All Western blot data represent at least three biological replicates with one to two technical replicates (*N*=5-8). Error bars represent s.d. and black points represent individual data points. *P<0.05, ***P<0.001, ****P<0.0001, ns=not significant.

To gain insight into how coilin is involved in m^6^A RNA methylation, we decided to first assess the gene expression of proteins that participate in the m^6^A regulatory network, and because we had a particular interest in miRNA biogenesis, we decided to focus on the *writer* METTL3 and the *reader* hnRNPA2B1. At this point, we included the KD of WRAP53β, another protein that is important for CB assembly (Mahmoudi et al., 2010), as a positive control against CB dysfunction. In HeLa cells, we found that coilin KD induced the RNA expression of METTL3 while WRAP53 KD had no effect. However, the KD of both coilin and WRAP53 reduced the RNA expression of hnRNPA2B1 (Fig. 2A). At the protein level, we found that coilin KD had the opposite effect on METTL3, suppressing protein expression (Fig. 2B), and likewise reducing hnRNPA2B1 (Fig. 2C). Meanwhile, WRAP53 KD in HeLa cells had no effect on the protein expression of METTL3 or hnRNPA2B1 (Fig. 2B,C). In WI38 cells, we found a similar effect at the mRNA level with coilin KD where METTL3 was induced while hnRNPA2B1 was reduced (Fig. 2D). However, in this cell type, we also found an induction of both METTL3 and hnRNPA2B1 RNA upon WRAP53 KD as well (Fig. 2D). At the protein level, we found that both coilin and WRAP53 KD suppressed the protein expression of METTL3 (Fig. 2E). Concurrently, coilin KD suppressed the protein expression of hnRNPA2B1 while WRAP53 KD continued to have no effect (Fig. 2F). We have confirmed that the siRNA targets in these data achieve an appropriate KD (Fig. S2). These results point toward a regulatory effect that coilin has on the expression of METTL3 and hnRNPA2B1. The induction at the RNA level coupled with the reduction at the protein level for METTL3 would suggest that coilin's effect on METTL3 could be via post-translational modification and the induced mRNA could be a compensatory effect. A coilin-mediated effect on the post-translational modification of proteins has been observed previously (Lett et al., 2021, 2023). Specifically, we have found that coilin KD reduces the phosphorylation of DGCR8 (Lett et al., 2021) and SUMOylation of DGCR8, SMN, and Sp100 (Lett et al., 2023). However, for hnRNPA2B1, while we cannot rule out any changes in post-translational modification for this protein, the reduced mRNA and protein expression as a result of coilin KD might suggest an effect at the level of transcription instead.

Our findings for WRAP53β were surprising. While WRAP53 KD in HeLa cells appeared to have little to no effect on the expression of METTL3, in WI38 cells the effects were more pronounced. As there has been a noted distinction between how the nuclei of immortalized cells and normal cells are organized (Kennedy et al., 2000), this discrepancy is possibly due to the primary cell type being more sensitive to nuclear disorganization. WRAP53 KD has been shown to disrupt CB formation and drive the localization of other CB proteins, such as coilin, into the nucleolus (Mahmoudi et al., 2010). It is feasible then, that if coilin is regulating METTL3 at the post-translational level, a WRAP53 KD-induced re-localization of coilin might exhibit similar effects to coilin KD. Collectively, these results point toward coilin being involved in the protein expression of METTL3 and hnRNPA2B1, but does not preclude other CB proteins. We find that WRAP53 has little to no effect on the expression of these proteins in a cancer cell line, but in a primary cell line, KD may induce protein level suppression of METTL3, suggesting that other CB proteins or possibly the CB as whole could play a role in the expression of these proteins.

### Ectopic METTL3 interacts with coilin and its phosphorylation is reduced upon coilin knockdown

**Fig. 3.**
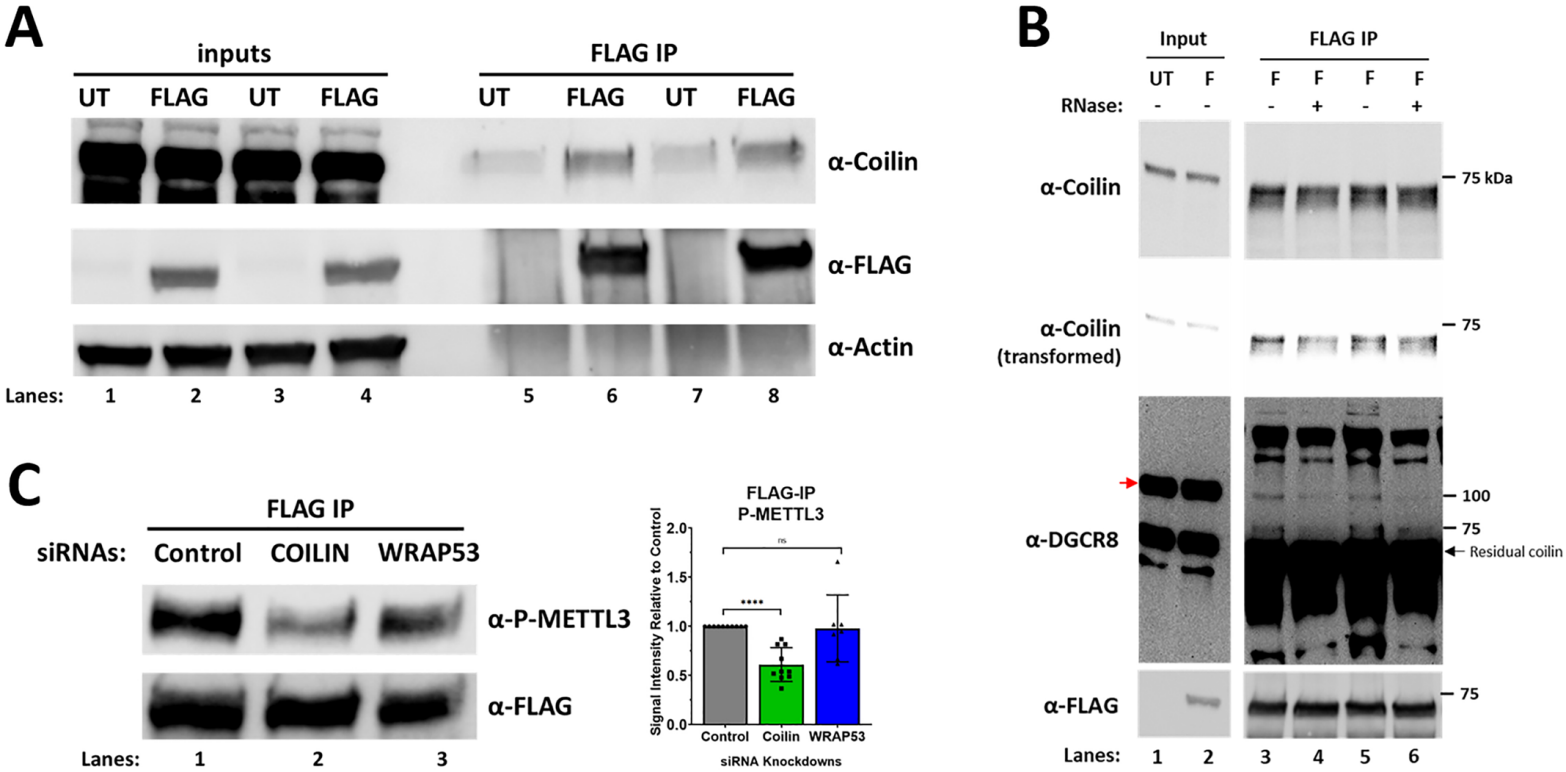
**METTL3 interacts with coilin and its nuclear stability and phosphorylation is reduced by coilin knockdown.** (A-B) FLAG-METTL3 was expressed in HeLa cells for 48 h. Protein lysates were collected and subjected to IP. (A) Immunoprecipitants and inputs were subject to Western blot to examine FLAG-METTL3, Coilin, and actin. Lanes 1-4 represent input samples. Lanes 5-8 represent immunoprecipitants. Lanes 1, 3, 5, and 7 represents samples collected from untransfected (UT) lysates. Lanes 2, 4, 6, and 8 represent samples collected from FLAG-METTL3 transfected lysates. (B) Immunoprecipitants were subject to RNase digestion prior to Western blot analysis of FLAG-METTL3, Coilin, and DGCR8. Red arrow denotes DGCR8. Lanes 1-2 represent input samples. Lanes 3-6 represent immunoprecipitants. Lanes 1 represents sample collected from UT lysates. Lanes 2-6 represent samples collected from FLAG-METTL3 transfected lysates. (C) siRNAs were transfected into HeLa cells for 48 h followed by expression of FLAG-METTL3 for 24 h. Protein lysates were collected and subjected to IP. Immunoprecipitants were subject to Western blotting to examine FLAG-METTL3 and phospho-Ser43 (P-METTL3). For quantification, signal intensities were determined for P-METTL3 and FLAG. A ratio of P-METTL3/FLAG was calculated and normalized to control with control set to 1. Data represent five biological replicates with one to two technical repeats (*N*=7-10). Error bars represent s.d. and black points represent individual data points. **P<0.01, ****P<0.0001, ns=not significant.

We have observed that coilin regulates the post-translational modification of DGCR8, SMN, and Sp100 (Lett et al., 2021, 2023). For each of these proteins, there exists an interaction, either direct or indirect, between the target protein and coilin. Thus, to understand if coilin might be playing a role in the post-translational modification of METTL3 our first goal was to investigate whether an interaction exists between METTL3 and coilin. To assess this, we ectopically expressed a FLAG-tagged METTL3 plasmid in HeLa cells and performed a FLAG immunoprecipitation (IP) followed by Western blotting. Upon probing for coilin (Fig. 3A), we found that coilin exhibited a small degree of nonspecific interaction in the untransfected IP (Lanes 5 and 7). However, upon FLAG-METTL3 expression and IP, we found that coilin was enriched over the background signal (Lanes 6 and 8). We next assessed whether the interaction between ectopic METTL3 and coilin was RNA-dependent. For this assay, we transfected HeLa cells with FLAG-METTL3 and performed a FLAG IP followed by RNase treatment and then subjected the immunoprecipitant to Western blotting. Upon probing for coilin (Fig. 3B), we found that RNase treatment (Lanes 4 and 6) partially reduced the interaction between FLAG-METTL3 and coilin when compared to no RNase treatment (Lanes 3 and 5). As a positive control, we also assessed the interaction between FLAG-METTL3 and DGCR8 as it has been reported that METTL3 and DGCR8 interact in a RNA-dependent manner (Alarcón et al., 2015a) and, as expected, we found that the RNase treatment also reduced interaction between FLAG-METTL3 and DGCR8. While potentially exciting, we do acknowledge that these data are limited by the ectopic expression of METTL3, which can result in nonspecific interactions. These data also suggest that coilin interaction with the METTL3 complex is likely transient, and the stringency of the IP buffer may impact this analysis. Nevertheless, these findings were quite interesting as a 2019 report (Ignatova et al., 2019) examined the interactome of METTL proteins and found that coilin interacted with several METTL proteins, but not METTL3. However, another major CB protein, the survival of motor neuron protein (SMN) (Sleeman et al., 2001; Young et al., 2000; Hebert et al., 2001; Sleeman et al., 2003), was observed to interact with METTL3, as well as several other METTL proteins. As SMN interacts highly with coilin (Sleeman et al., 2001; Young et al., 2000; Hebert et al., 2001; Sleeman et al., 2003), it is possible that an indirect, RNA-dependent and transient interaction exists between METTL3 and coilin that may be facilitating changes at the post-translational level.

We have published that coilin plays a role in phosphorylation of DGCR8 (Lett et al., 2021) and the phosphorylation of METTL3 has been found to regulate protein stability (Sun et al., 2020). Thus, we were interested in examining if coilin influenced the phosphorylation of METTL3. We knocked down coilin or WRAP53 and then co-transfected FLAG-METTL3 after which we performed an IP of FLAG-METTL3 and subjected the immunoprecipitant to Western blotting where we examined METTL3 phosphorylation using an antibody that recognizes phosphorylation of Serine 43. When normalized to control, we found that coilin KD yielded a reduction in the phosphorylation of Serine 43 of FLAG-METTL3 while no consistent effect is observed with WRAP53 KD (Fig. 3C). This reinforces our earlier observation that the KD of coilin, but not WRAP53, in HeLa cells reduced the protein expression of METTL3. We next attempted to extend this analysis to endogenous METTL3, but unfortunately found that the phospho-METTL3 antibody does not interact well with endogenous METTL3 (Fig. S3A) and could only strongly be detected upon ectopic expression and IP (Fig. S3B). We acknowledge that our assessment of METTL3 phosphorylation is limited by the lack of endogenous analysis. Future studies on coilin-mediated METTL3 phosphorylation should be focused on endogenous METTL3 and should expand beyond the single Serine 43 residue.

Collectively, these findings suggest that coilin may interact with METTL3 and help facilitate its phosphorylation. While coilin is not a kinase, it is possible that coilin's nature as a scaffolding protein could aid in bringing METTL3 and its associated kinase within closer proximity, facilitating an efficient phosphorylation. ERK has been recognized as a kinase that phosphorylates METTL3 (Sun et al., 2020). ERK has two binding domains that facilitate its interaction with other proteins: the ERK common docking domain (CD) (Matsuoka et al., 2007) or the ERK F-site recruitment site (FRS) (Roskoski, 2012). Analysis using the Eukaryotic Linear Motif database (http://elm.eu.org) revealed that coilin possesses several predicted CD domains and one predicted F-site that could potentially facilitate association with ERK. This is in comparison to the one predicted CD domain of SMN.

### Coilin knockdown decreases nuclear localization of METTL3 and disrupts the METTL3-METTL14-WTAP complex

**Fig. 4.**
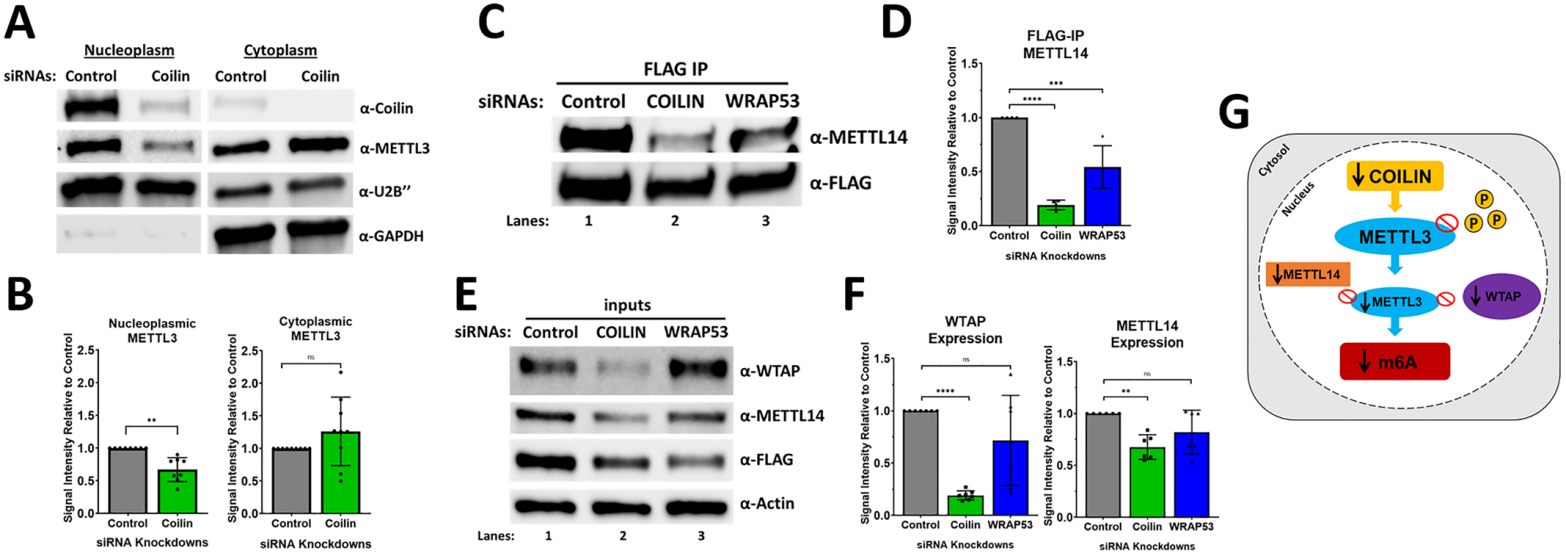
**Coilin knockdown decreases the protein expression of WTAP and METTL14 and disrupts METTL3-METTL14 complex.** (A-B) siRNAs were transfected into HeLa cells for 72 h followed by cell fractionation. Lysates collected from either fraction were subject to Western blot to examine coilin, METTL3, U2B‘‘ (nucleoplasmic marker), and GAPDH (cytoplasmic marker). For quantification, signal intensities were determined for METTL3, U2B‘‘, and GAPDH. A ratio of METTL3/U2B‘‘ (nucleoplasmic) or METTL3/GAPDH (cytoplasmic) was calculated and normalized to control with control set to 1. Data represent three biological replicates with two to three technical repeats (*N*=8-9). (C-F) siRNAs were transfected into HeLa cells for 48 h followed by expression of FLAG-METTL3 for 24 h. Protein lysates were collected and subjected to IP. Immunoprecipitants and inputs were subject to Western blot to examine FLAG-METTL3, METTL14, WTAP, and Beta Actin. For analysis of METTL3 interactions, signal intensities were determined for METTL14 and FLAG. A ratio of METTL14/FLAG was calculated and normalized to control with control set to 1. Data represent four biological replicates (*N*=4). For analysis of METTL14 and WTAP expression, signal intensities were determined for the targets and Beta-Actin. A target/actin ratio was calculated and normalized to control with control set to 1. Data represent four biological replicates with one to two technical repeats (*N*=7). Error bars represent s.d. and black points represent individual data points. ***P<0.001, ****P<0.0001, ns=not significant. (E) Model illustrating the proposed coilin regulatory effect on METTL3 and m^6^A RNA methylation.

Because coilin is a strictly nuclear protein while METTL3 can be found in both the nucleoplasm and cytoplasm (Lin et al., 2016; Schöller et al., 2018; Wei et al., 2022), we next wanted to observe if the coilin-mediated reduction of METTL3 was specific to either compartment of the cell. For this, we knocked down coilin and conducted a cell fractionation procedure to extract protein lysate enriched in either the nucleoplasm or cytoplasm. This lysate was then subjected to Western blotting, where we examined the localization of METTL3 while using U2 snRNA protein B‘‘ (U2B‘‘) as a marker for the nucleoplasmic fraction and glyceraldehyde 3-phosphate dehydrogenase (GAPDH) as a marker for the cytoplasmic fraction. We found that coilin KD yielded a decrease in METTL3 localized within the nucleoplasm (Fig. 4A,B). While there was a trending increase of METTL3 within the cytoplasmic fraction, we did not observe a consistent effect due to coilin KD (Fig. 4A,B).

Our previous findings prompted us to extend our analysis of coilin's interaction with METTL3 to the greater METTL3-METTL14-WTAP complex. It is understood that this complex functions with METTL3 as the catalytic component that facilitates methyltransferase activity while METTL14 and WTAP act as cofactors that aid in the specificity of RNA binding and recruitment into nuclear speckles, respectively (Schöller et al., 2018). To test this, we conducted a KD of coilin or WRAP53 and co-transfected FLAG-METTL3. We collected protein lysates from these KDs and conducted an IP of FLAG-METTL3, after which immunoprecipitates were subjected to Western blotting analysis targeting METTL14 (Fig. 4C). Upon probing for METTL14, we found that coilin KD reduced the interaction between FLAG-METTL3 and METTL14 (Fig. 4D). Surprisingly, WRAP53 KD also pointed toward a decreased FLAG-METTL3 and METTL14 interaction (Fig. 4D). We next aimed to validate this result in a purely endogenous system by knocking down coilin or WRAP53, collecting lysates from these cells, and performing an IP of endogenous METTL3 (Fig. S4). Interestingly, in the purely endogenous system, we observed that only coilin KD appeared to impair METTL3-METTL14 interaction (Fig. S4 Lanes 5 and 6 compared with Lane 4). We next used input samples from the FLAG IP to examine the protein levels of METTL14 and WTAP (Fig. 4E). Here, we found that coilin KD, but not WRAP53 KD, induces a drastic suppression of WTAP and a minor suppression of METTL14 at the protein level (Fig. 4F).

We believe that coilin might be influencing the disruption of the METTL3–METTL14 complex through phosphorylation. Inhibiting phosphorylation of METTL3 has been shown to decrease binding affinity to WTAP, which aids in localization of the METTL3 to nuclear speckles and overall nuclear retention (Schöller et al., 2018). However, phosphorylation has not been shown to influence binding affinity with METTL14 and to our knowledge, there is currently nothing in the literature that describes how interactions between METTL3 and METTL14 may be modulated. A possible explanation, however, is that while METTL3 can reside in both the cytoplasm and nucleoplasm, METTL14 is a nuclear protein (Lin et al., 2016). Due to a nuclear environment in which METTL3 residence is downregulated, when we IP FLAG-METTL3, we are likely enriching for the cytoplasmic portion where METTL14 is significantly less abundant. As for the protein level expressions of METTL14 and WTAP, it has been shown that decreased phosphorylation of WTAP also decreases its stability and it is reasonable to suggest that coilin's KD may also be influencing WTAP phosphorylation. Future studies observing coilin's potential role in WTAP phosphorylation is an exciting avenue for our lab. While METTL14 has also been noted to be phosphorylated (Schöller et al., 2018), there is no evidence to suggest that this modification directs stability. However, it has been observed that METTL3 protects METTL14 from degradation (Zeng et al., 2023), and in an environment in which METTL3 is suppressed, METTL14 is subject to protein degradation. Future work will be needed to examine how WRAP53 is participating in the disruption of the METTL3-METTL14 complex.

While we cannot rule out added influences from other CB proteins or possibly the whole CB as mediators in m^6^A RNA methylation, this work reports a novel finding for coilin in mediating m^6^A and opens doors for future studies to interrogate how coilin or other CB proteins influence the m^6^A regulatory network. Collectively, we find that reducing coilin expression results in suppression of the nucleoplasmic portion of METTL3, decreased phosphorylation of ectopic METTL3, decreased METTL3-METTL14 interaction, and suppressed expression of hnRNPA2B1, METTL14, and WTAP. This all summates to a suggestive influence of coilin on the m^6^A RNA methylation of various RNAs, but specifically pri-Let7a and pri-miR-21 (Fig. 4G).

## MATERIALS AND METHODS

### Cell lines, plasmids, and transfections

HeLa and WI-38 cell lines were obtained from the American Type Culture Collection (ATCC). These lines are routinely authenticated by assessing their morphology and nuclear organization (WI-38 cells have very few CBs compared to HeLa cells). Cells were cultured in DMEM media containing 10% fetal calf serum in a 5% CO_2_ incubator at 37°C. All siRNAs were obtained from Integrated DNA Technologies (IDT) (Coralville, IA, USA) and used with RNAiMax (Invitrogen, Carlsbad, CA, USA) per the manufacturer's protocol. Negative control siRNAs were supplied by IDT and a description of all other siRNAs can be found below. siRNA transfections were conducted for at least 72 h and, if required, an additional transfection was conducted to extend KD period to 96 or 120 h. DNA transfections in HeLa cells were conducted using FuGene HD (Promega, Madison, WI, USA) according to the manufacturer's protocol. FLAG-tagged METTL3 plasmid was obtained from Addgene (Watertown, MA, USA) under plasmid #160250. FLAG-METTL3 transfections were conducted for 24 or 48 h.

**Table:** 

List of siRNAs	
Duplex name	Duplex sequences (5′ to 3′)
Coilin #1	Forward (F): GAGAGAACCUGGGAAAUUUUU
	Reverse (R): AAAUUUCCCAGGUUCCUCUU
Coilin #2	F: GACCCAGCAAGUAGAUAUAGAAATT
	R: AAUUUCUAUAUCUACUUGCUGGGUCUC
METTL3	F: CUAAACCUGAAGAGUGAUAUUUGTA
	R: UACAAAUAUCACUCUUCAGGUUUAGCU
WRAP53	F: GGUGAUACCAUCUAUGAUUACUGCT
	R: AGCAGUAAUCAUAGAUGGUAUCACCUU

### m^6^A RNA dot blot assay

HeLa cells were transfected with negative control or coilin siRNA for 96 h. Total RNA was next extracted with TRI-REAGENT (Molecular Research Center), according the manufacturer's protocol. 500 ng of total RNA is collected in a volume of no more than 2 μl in a 1.5 ml tube and incubated at 95°C for 5 min. RNA is next chilled on ice before being dotted onto a BrightStar Plus positively charged nylon membrane (Invitrogen). The membrane is next subjected to a UV cross-linker (UVP, Upland, CA, USA) at a setting of 120,000 μJ/cm^2^. After which, the membrane is briefly rinsed in TBS-T (50 mM Tris, 150 mM NaCl, 0.05% Tween20) and then blocked with a solution of 5% non-fat milk in TBS-T for 1 h. After blocking, the membrane is then immunoblotted overnight with m^6^A antibody (Synaptic Systems) in a solution of 2.5% non-fat milk in TBS-T. To detect dots, the membrane was washed three times for 10 min each with TBS-T and next incubated with a species-specific HRP-conjugated antibody in 2.5% non-fat milk for 1 h followed three more washes in TBS-T and finally a 5 min incubation with SuperSignal West Pico Chemiluminescent substrate (ThermoFisher Scientific). After immunoblotting, the membrane was then incubated methylene blue staining buffer (0.2% methylene blue in 0.4 M sodium acetate and 0.4 M acetic acid) for 30–60 min and washed several times with distilled water to assess total RNA loading. Imaging was done on a ChemiDoc (BioRad, Hercules, CA, USA) with QuantityOne software. Adjustments to images were made using the transformation settings on QuantityOne software and applied across the entire image. Dots were quantified using QuantityOne software. Methylene Blue was used to normalize signal intensity of m^6^A for each dot.

### m^6^A RNA immunoprecipitation (meRIP)

HeLa or WI38 cells were transfected with negative control, coilin #1, coilin #2, METTL3, or WRAP53 siRNAs for 72 or 96 h. After which, total RNA was extracted with TRI-REAGENT. 5 μg of total RNA is next incubated in a solution of RNA IP Reaction Buffer (150 mM NaCl, 10 mM Tris-HCl, pH 7.5, 0.1% NP-40 in nuclease free H_2_O) with 3 μg of m^6^A antibody (Synaptic Systems) for 30 min while rocking at 4°C. Next, 30 μl of 50% Protein G Sepharose 4 Fast Flow beads (GE Healthcare) is added and incubated for 2 h while rocking at 4°C. The antibody-bead complexes were next washed three times with RNA IP Reaction Buffer before aspirating the beads dry. RNA is eluted from with beads using TRI-REAGENT supplemented with GlycoBlue coprecipitant. Finally, meRIP RNA is subjected to quantitative real-time PCR.

### Quantitative real-time PCR

RNA from HeLa and WI-38 cells was extracted with TRI-REAGENT (Molecular Research Center, Cincinnati, OH, USA) according to the manufacturer‘s protocol. Reactions were set up with 50 ng total RNA in Brilliant II SYBR Green qRT-PCR master mix (Agilent, Santa Clara, CA, USA) using an Agilent MX3000P qRT-PCR system. Oligonucleotides used were obtained from Integrated DNA Technologies (Coralville, IA, USA). A complete list of primers can be found below. For mRNA analysis, GAPDH served as the normalizer. For meRIP analysis, 18 s rRNA served as the normalizer. The primers used for primary let7a and miR-21 do not overlap with the precursor miRNA sequences, so only the primary transcript is amplified. Amplification rates, Ct values. and dissociation curve analyses of products were determined using MxPro (version 4.01) software. Relative expression was determined using the 2−ΔΔCT method (Livak and Schmittgen, 2001). GraphPad Prism was used for *post-hoc* statistical analysis and for histogram generation.

**Table:** 

List of qRT-PCR primers (5′ to 3′)		
18 s rRNA	F: GAGAAACGGCTACCACATCCA
	R: CGGGTCGGGAGTGGGTAATTT
Primary Let7a	F: GATTCCTTTTCACCATTCACCCTGGATGTT
	R: TTTCTATCAGACCGCCTGGATGCAGACTTT
Primary miR-21	F: CGGGTAGCTTATCAGACTGATGTTGAC
	R: CACCAGACAGAAGGACCAGAGTTTCTG
GAPDH	F: GACTCATGACCACAGTCCATGCCATC
	R: CCACAGCCTTGGCAGCGCCAGTAGAGG
METTL3	F: GAGAGCCTTCTGAACCAACAGTCC
	R: CCCGACCTCGAGAGCGAAAT
hnRNPA2B1	F: AATTGATGGGAGAGTAGTTGAGCCAAA
	R: TCCTCAGTATCTTCTTTAATTCCGCCAAC
Coilin	F: CTTGAGAGAACCTGGGAAATTTG
	R: GTCTGGGGTCAATCAACTCTTTCC
WRAP53	F: CCCCGATGAATAAAAATGCGG
	R: GGGAACCAAACTCTGTTTCCAGGG

### Immunoprecipitation (IP)

For coilin and FLAG-METTL3 interactions, HeLa cells were plated in a 100 mm dish, transfected with 3 μg FLAG-METTL3 for 48, and lysed in RIPA buffer (50 mM Tris HCl pH 7.6, 150 mM NaCl, 1% NP-40, 0.25% Na-Deoxycholate, 1 mM EDTA, 0.1% SDS) plus protease inhibitor cocktail (ThermoFisher Scientific). For FLAG-METTL3 interactions with METTL14 or DGCR8, HeLa cells were plated in a 60 mm dish, transfected with 500 ng FLAG-METTL3 for 48, and cells were lysed in IP lysis buffer (25 mM Tris HCl pH 7.4, 150 mM NaCl, 1 mM EDTA, 1% NP-40, 5% glycerol) plus protease inhibitor cocktail. Lysates were sonicated three times with a ThermoFisher Scientific sonic dismembrator (Model 100) for 5 s each using the output setting of 1 and finally centrifuged at 12,000 rpm for 15 min at 4°C. For FLAG-METTL3 transfections, transfected and untransfected lysates were incubated with 20 µl anti-FLAG-M2 affinity agarose beads (Sigma-Aldrich, St. Louis, MO, USA) for 2 h. For endogenous METTL3 IP, lysates were incubated with 1 μg of METTL3 or IgG for 2 h while rocking at 4°C before adding 30 μl 50% Protein G Sepharose 4 Fast Flow beads (GE Healthcare) and incubating for 2 h while rocking at 4°C. Immunoprecipitates were washed three times with RIPA or IP lysis buffer followed by analysis via Western blotting. For RNase digestion, after washing immunoprecipitates and aspirating beads dry, beads were resuspended in 40 μl of PBS supplemented with or without 1 μl of RNase A/T1 Cocktail Enzyme Mix (Invitrogen) and incubated at 37°C for 30 min. After incubation, immunoprecipitates were washed twice with PBS followed by analysis via Western blotting.

### Western blotting

For standard Western blotting analysis, HeLa or WI38 cells were lysed in RIPA buffer. Lysates were sonicated three times with a Fisher Scientific sonic dismembrator (Model 100) for 5 s each using the output setting of 1 and finally centrifuged at 12,000 rpm for 15 min at 4°C. Lysate (15 μl) was run on a precast 7.5 or 10% Mini-Protean Gel (Bio-Rad Laboratories, Hercules, CA, USA). For nuclear and cytoplasmic extractions, HeLa cells were collected using NE-PER Nuclear and Cytoplasmic Extraction Reagents (ThermoFisher Scientific) according to manufacturer's protocol and samples were run on a 10% Mini-Protean gel. For IP experiments described above, 20 µl of 2X SDS loading buffer was added to aspirated beads. Immunoprecipitates and input lysates were run on a precast 7.5% Mini-Protean Gel (Bio-Rad Laboratories, Hercules, CA, USA). Western transfer and detection were conducted as previously described (Poole and Hebert, 2016). A complete list of all antibodies used can be found below. Secondary antibodies used were goat anti-mouse HRP or goat anti-rabbit HRP. Bands were detected with SuperSignal West Pico Chemiluminescent Substrate (ThermoFisher Scientific) following the manufacturer's suggested protocol. Imaging was done on a ChemiDoc (BioRad) with QuantityOne software. Adjustments to images were made using the transformation settings on QuantityOne software and applied across the entire image. Bands were quantified using QuantityOne software. Beta-actin was used to normalize signal intensity for standard Western blot samples, FLAG was used to normalize signal intensity in FLAG IP samples, U2B‘‘ was used to normalize signal intensity in nucleoplasmic samples, and GAPDH was used to normalized signal intensity in cytoplasmic samples. GraphPad Prism was used for *post-hoc* statistical analysis and for histogram generation.

**Table:** 

List of primary antibodies used		
Primary antibody	Source
Anti-Coilin	#sc-32860, Santa Cruz Biotechnology, Dallas, TX, USA
Anti-WRAP53	#A301-442A, Bethyl Laboratories, Montgomery, TX, USA
Anti-METTL3 (mouse)	#H00056339-B01P, Novus Biologicals, Centennial, CO, USA
Anti-METTL3 (rabbit)	#ab240595, Abcam, Waltham, MA, USA
Anti-Phospho-METTL (Serine 43)	#ABE2611, MilliporeSigma, Burlington, MA, USA
Anti-hnRNPA2B1	#NB120-6102, Novus Biologicals, Centennial, CO, USA
Anti-FLAG	#F3165, Sigma Aldrich, St. Louis, MO, USA
Anti-WTAP	#10200-1-AP, Proteintech, Rosemont, IL, USA
Anti-METTL14	#26158-1-AP, Proteintech, Rosemont, IL, USA
Anti-U2B‘‘	#57036, MP Biomedicals, Aurora, OH, USA
Anti-GAPDH	#5174, Cell Signaling Technology, Danvers, MA, USA
Anti-Beta Actin	#3700, Cell Signaling Technology, Danvers, MA, USA
Anti-m^6^A	#202011, Synaptic Systems, Goettingen, Germany

### Statistical analysis

GraphPad Prism was used for all *post-hoc* statistical analysis. Analyses, first, consisted of a Shapiro–Wilk test to determine normality in the collected data from each experiment. Assuming all datasets had a normal distribution and did not violate an assumption for the ANOVA, the data is then subjected to a one-way ANOVA with a Dunnett's multiple comparison test, comparing only KD datasets to control datasets. If the collected data has a normal distribution, but violated an assumption of the ANOVA, the data is then analyzed by paired *t*-test (parametric *t*-test). If the collected data does not have a normal distribution and violates an assumption of ANOVA, data is analyzed by the Wilcoxon matched-pairs signed rank test (nonparametric *t*-test). *P* values recorded from each of the previously named tests are reported in the figure legends.

## Supplementary Material

10.1242/biolopen.060116_sup1 Supplementary information

Table S1. Raw PCR data
