# Supplementary material for: Coilin mediates m6A RNA methylation through phosphorylation of METTL3
Source: Biol Open. 2023 Dec 5;12(12):bio060116. doi: 10.1242/bio.060116 (PMC10714142; doi:10.1242/bio.060116)
Supplement: Supplementary information [file biolopen-12-060116-s1.pdf]

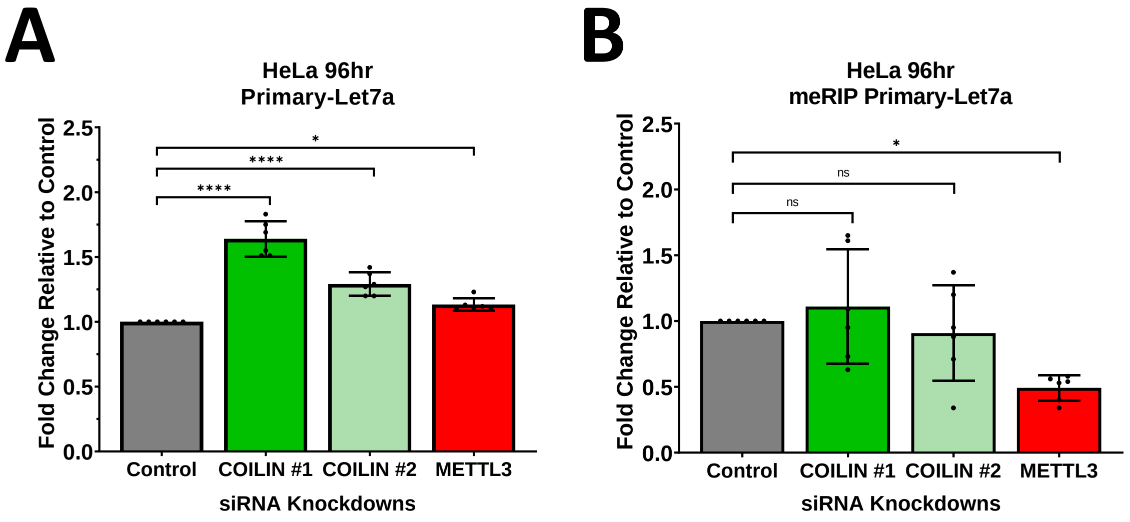

Fig. S1. Coilin KD for 96 h is not sufficient to reduce m^{6}A modification in pri-Let7a. siRNAs were transfected into HeLa cells for 96 h (A-B). Total RNA was collected and 5 ug was used for meRIP using anti m^{6}A followed by a second RNA extraction. RNAs were subject to qRT-PCR. Data represents 3 biological replicates with 2-3 technical repeats (N = 6-9). Error bars represent SD and black points represent individual data points. *$p < 0.05$, ****$p < 0.0001$, ns = not significant.

Biology Open • Supplementary information

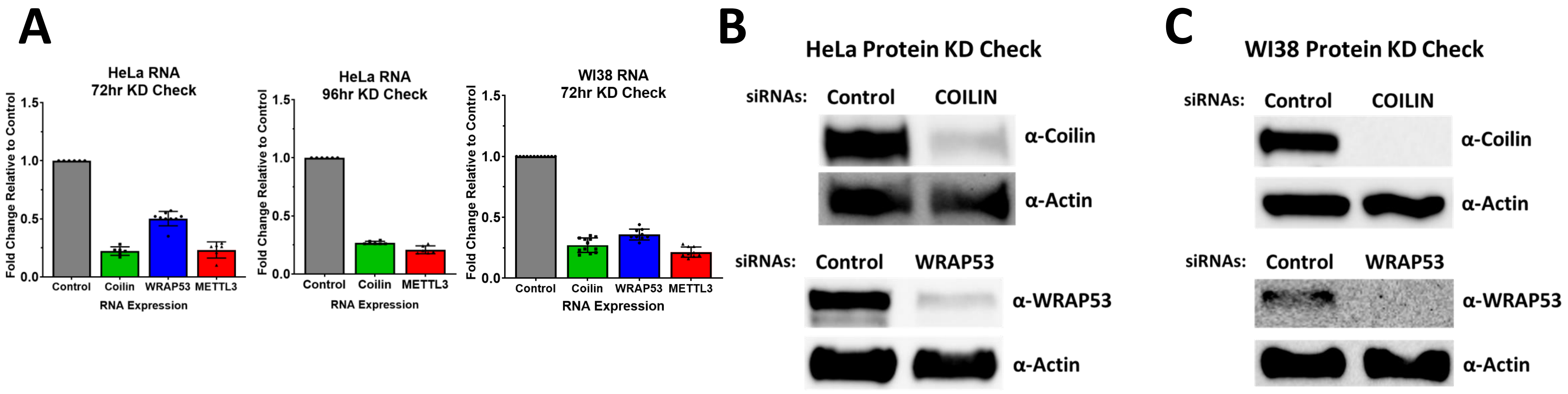

Fig. S2. Assessment of knockdown efficiency in RNA and protein samples. A) qRT-PCR analysis of the RNA expression Coilin, WRAP53, or METTL3 after 72 or 96 h KD in HeLa or WI38 cells. Targets included GAPDH, Coilin, WRAP53, and METTL3. Data represents 2-4 biological replicates with 3 technical repeats (N = 6-12). Error bars represent SD and black points represent individual data points. B-C) Western blot analysis of coilin or WRAP53 protein expression after 72 h KD in HeLa cells or WI38 cells.

Biology Open • Supplementary information

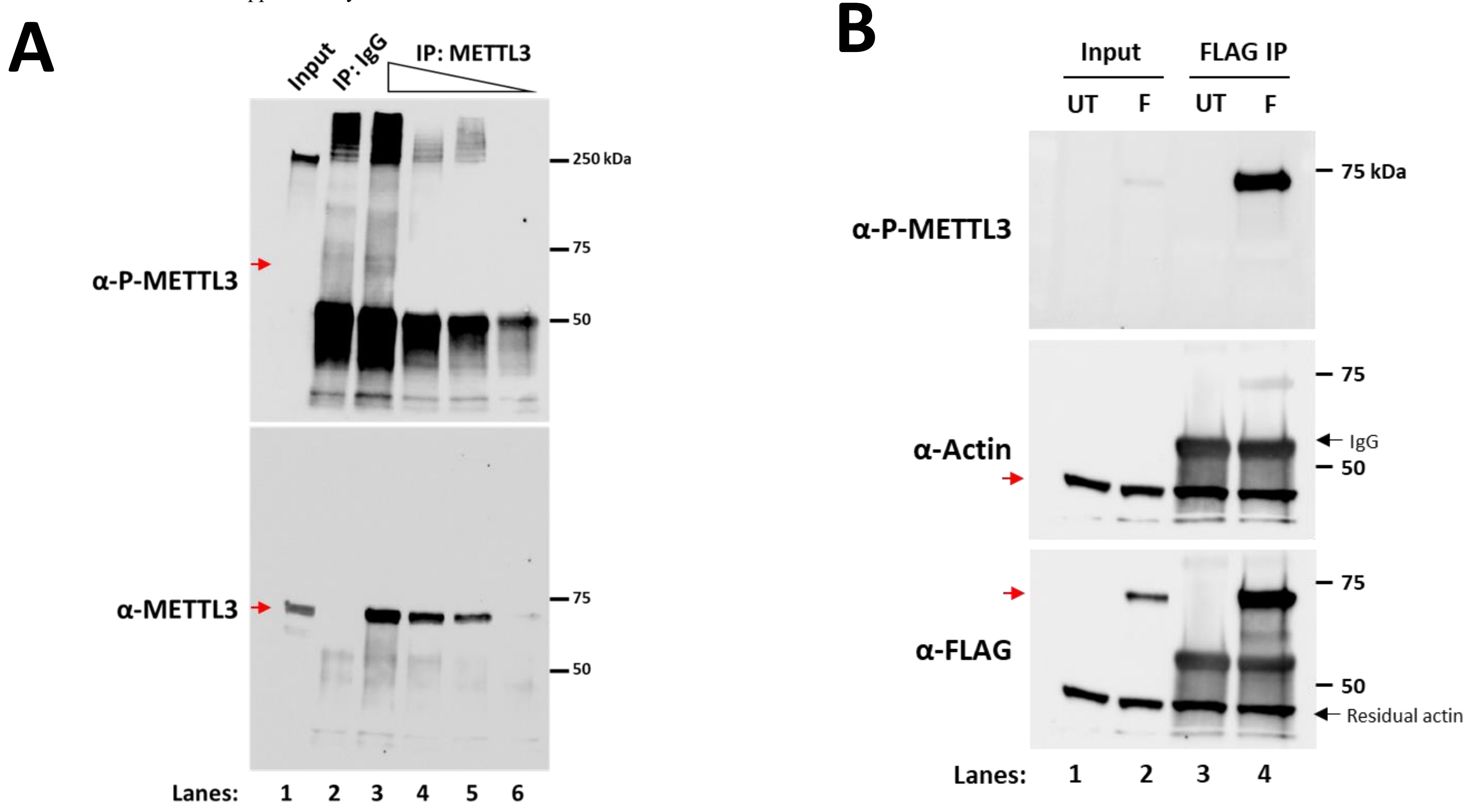

Fig. S3. Phospho-METTL3 antibody is effective only in ectopic METTL3. A) HeLa protein lysates were collected and subjected to IP with METTL3 or IgG. Immunoprecipitants and inputs were subject to western blot to examine P-METTL3 and METTL3. Lane 1 represents input lysate. Lane 2 represents IgG IP. Lanes 3-6 represent METTL3 IPs with decreasing concentrations of METTL3 antibody. B) HeLa cells were transfected with FLAG-METTL3 for 24 h and protein lysates were collected and subjected to FLAG IP. Immunoprecipitants and inputs were subject to western blot to examine P-METTL3, FLAG-METTL3, and beta actin. Red arrows denote expected region for protein signal.

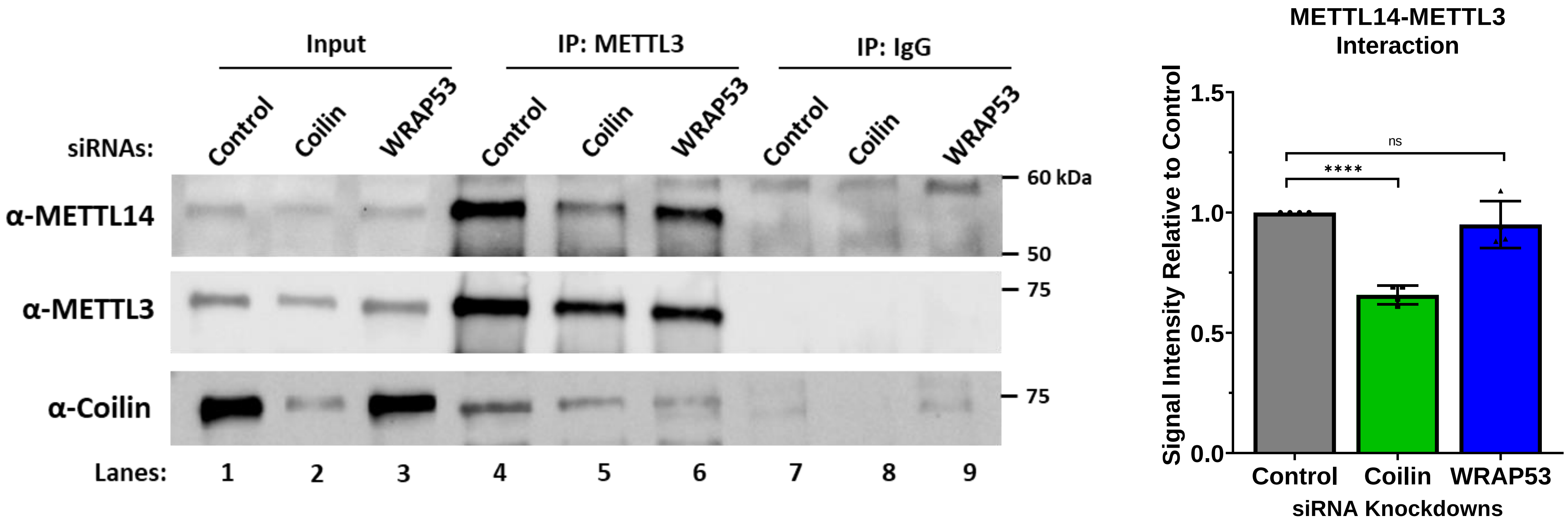

Fig. S4. Coilin suppression disrupts endogenous METTL3-METTL14 interaction. A) siRNAs were transfected into HeLa cells for 72 h. Protein lysates were collected and subjected to IP with METTL3 or IgG. Immunoprecipitants and inputs were subject to western blot to examine METTL3, METTL14, and coilin. For analysis of METTL3 interactions, signal intensities were determined for METTL14 and METTL3. A ratio of METTL14/METTL3 was calculated and normalized to control with control set to 1. Data represents 4 biological replicates (N = 4). Error bars represent SD and black points represent individual data points. ****$p < 0.0001$, ns = not significant.

Biology Open • Supplementary information

Biology Open (2023): doi:10.1242/bio.060116: Supplementary information

Figure 1A

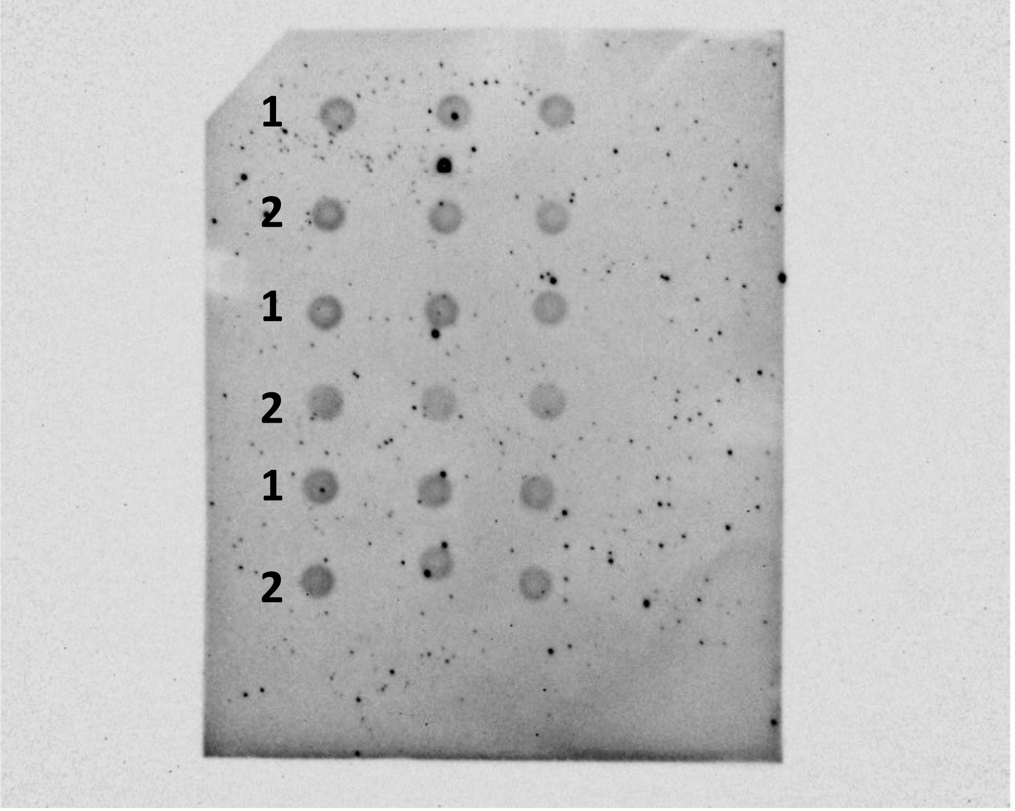

α-m6A

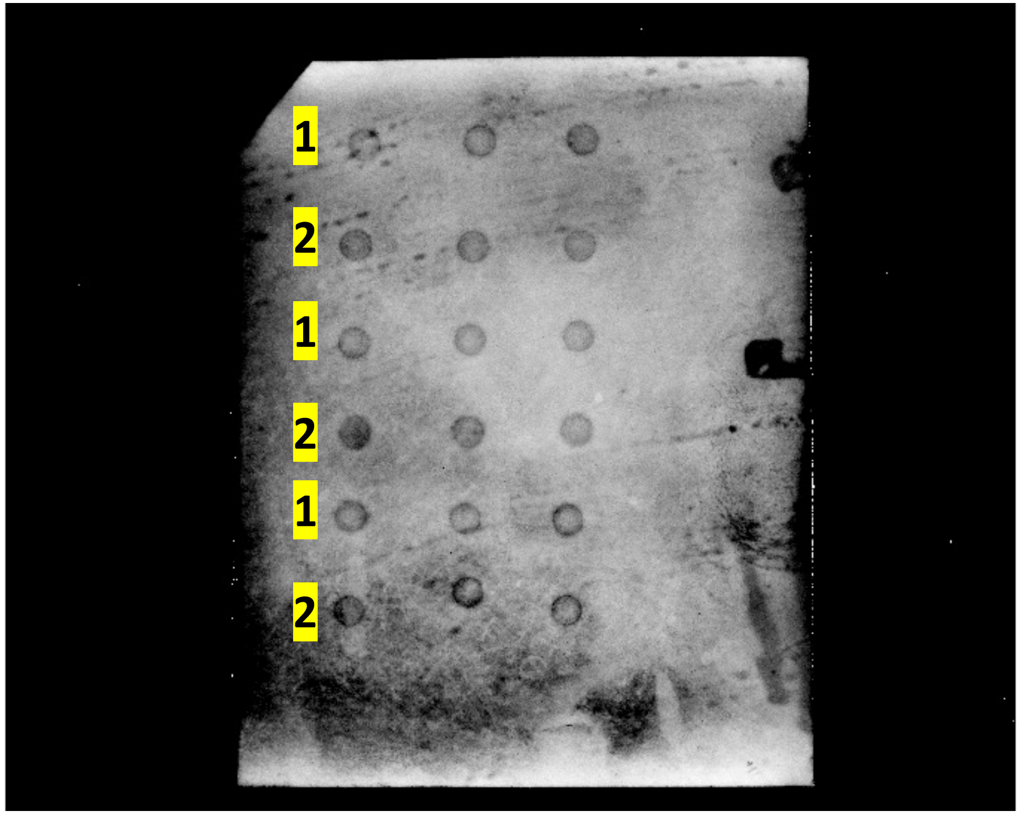

Methylene Blue

1 – Control KD
2 – Coilin KD

Biology Open • Supplementary information

Figure 2B

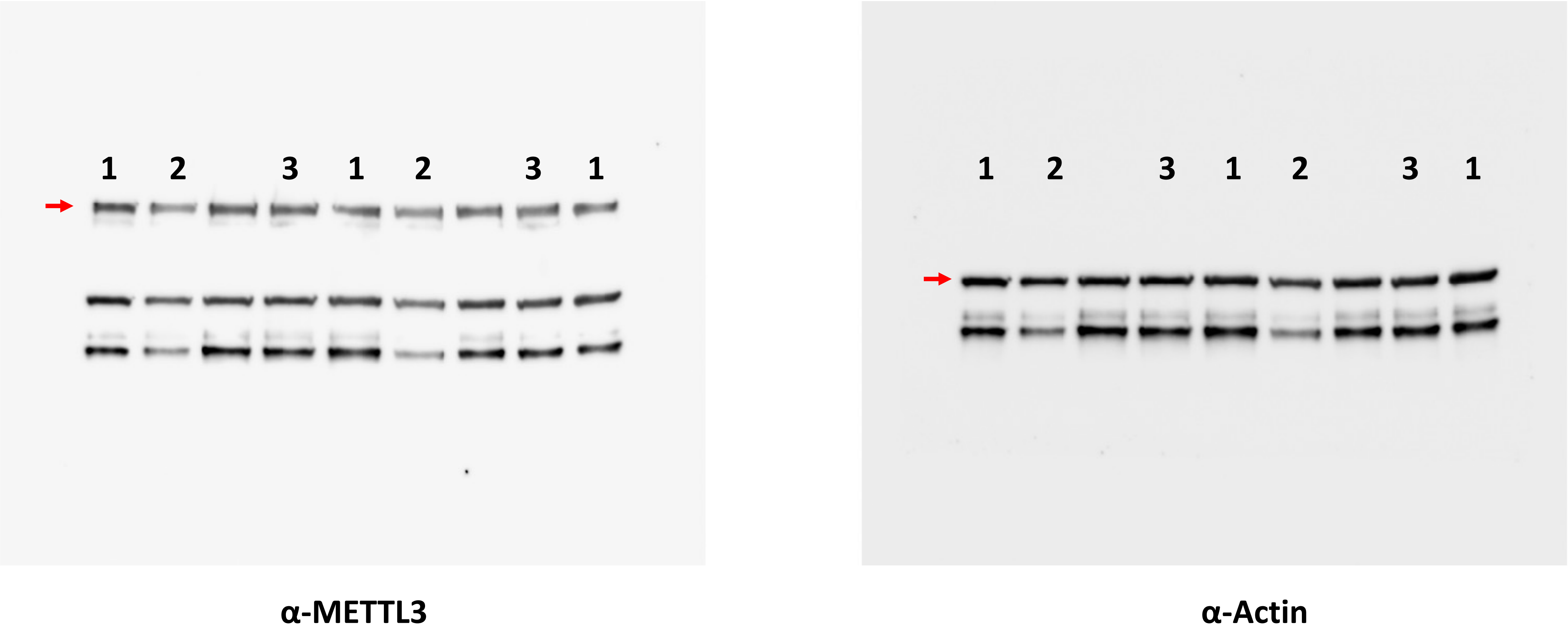

1 – Control KD
2 – Coilin KD
3 – WRAP53 KD

Biology Open • Supplementary information

Figure 2C

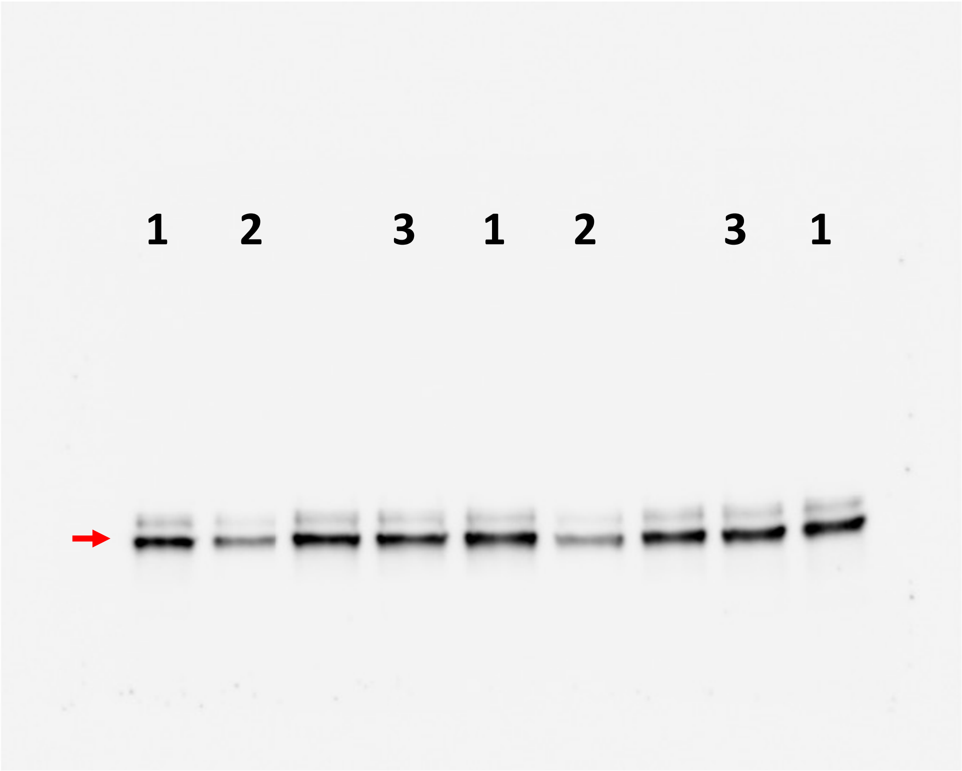

α-A2B1

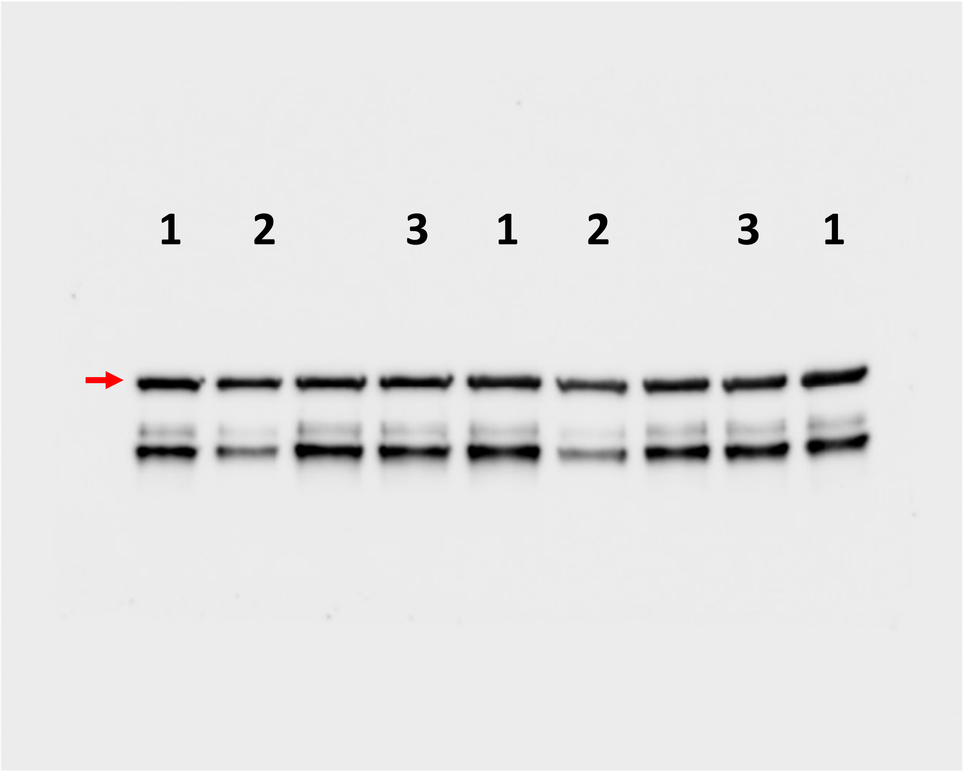

α-Actin

1 – Control KD
2 – Coilin KD
3 – WRAP53 KD

Biology Open • Supplementary information

Figure 2E

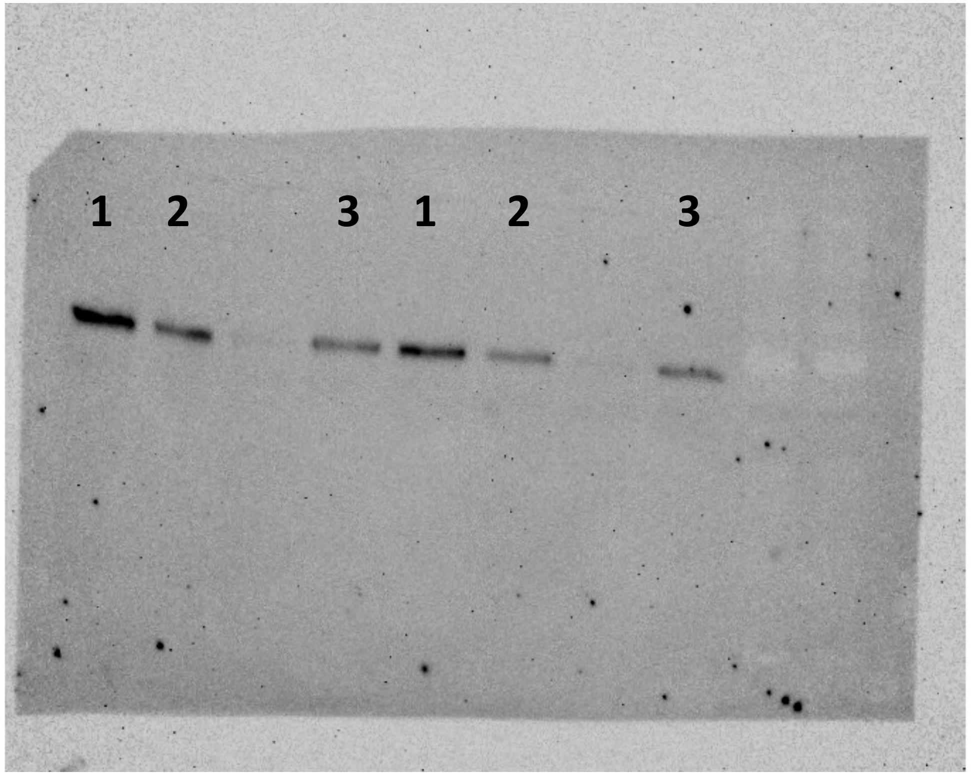

α-METTL3

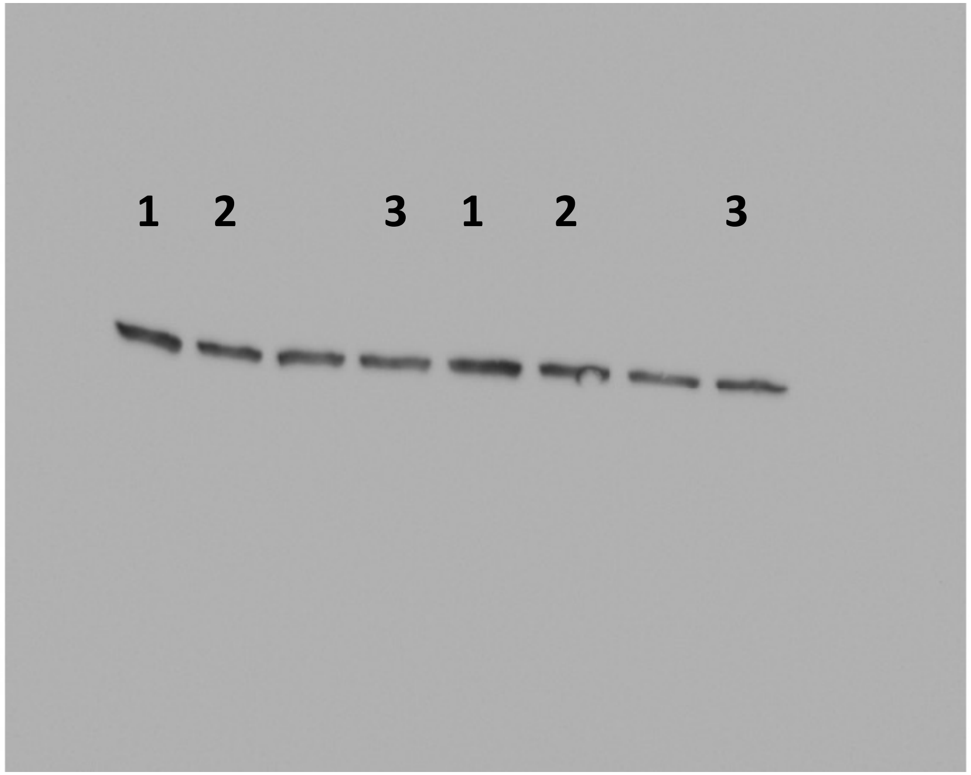

α-Actin

1 – Control KD
2 – Coilin KD
3 – WRAP53 KD

Biology Open • Supplementary information

Figure 2F

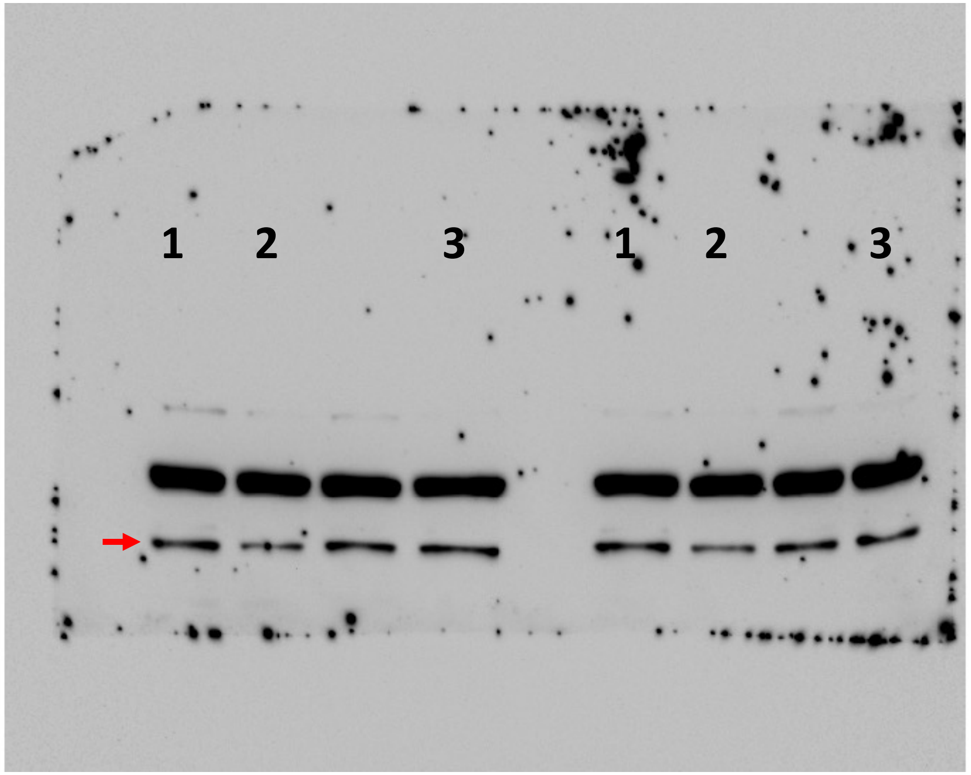

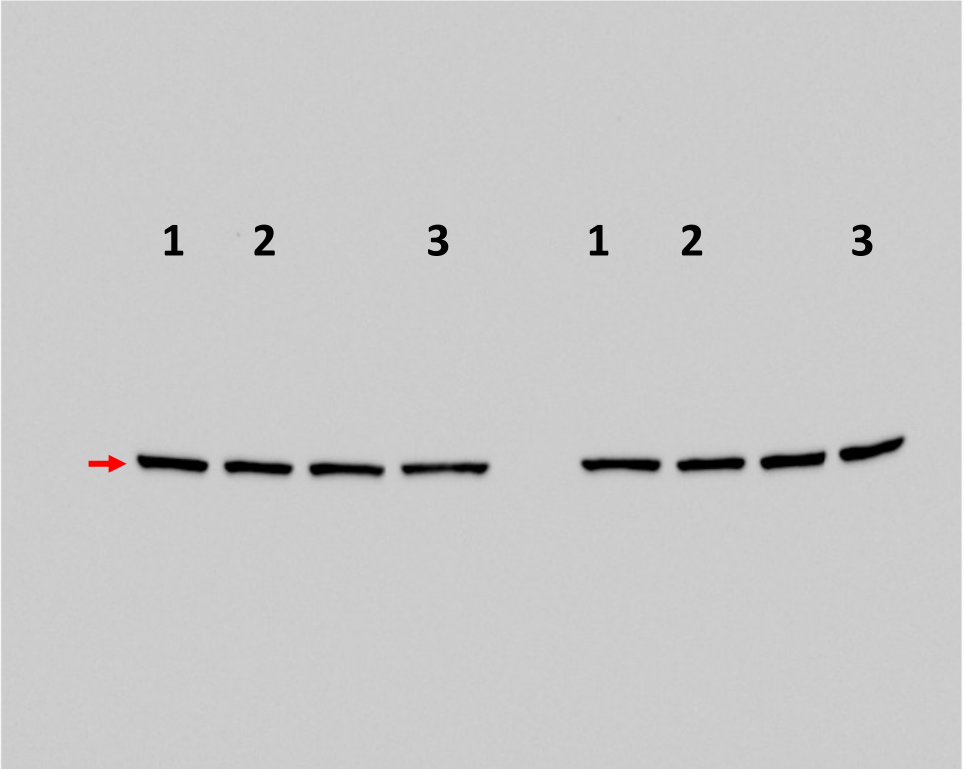

1 – Control KD
2 – Coilin KD
3 – WRAP53 KD

Biology Open • Supplementary information

Figure 3A

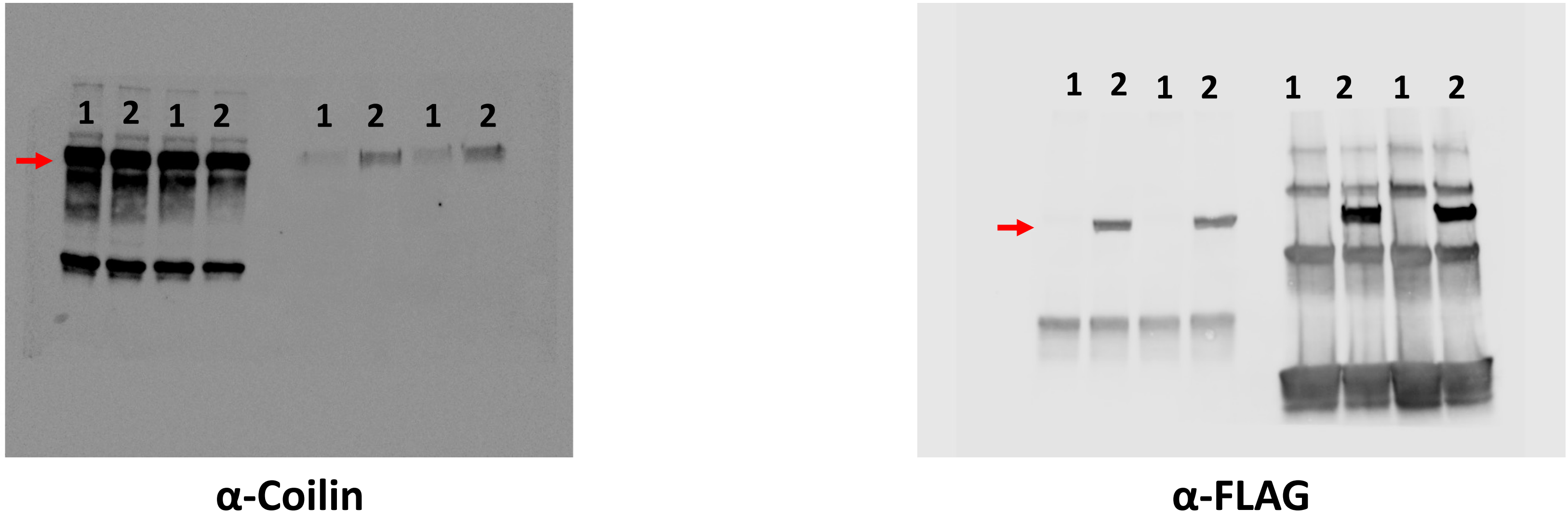

1 – Untransfected
2 – FLAG-METTL3 transfected

Biology Open • Supplementary information

Figure 3B

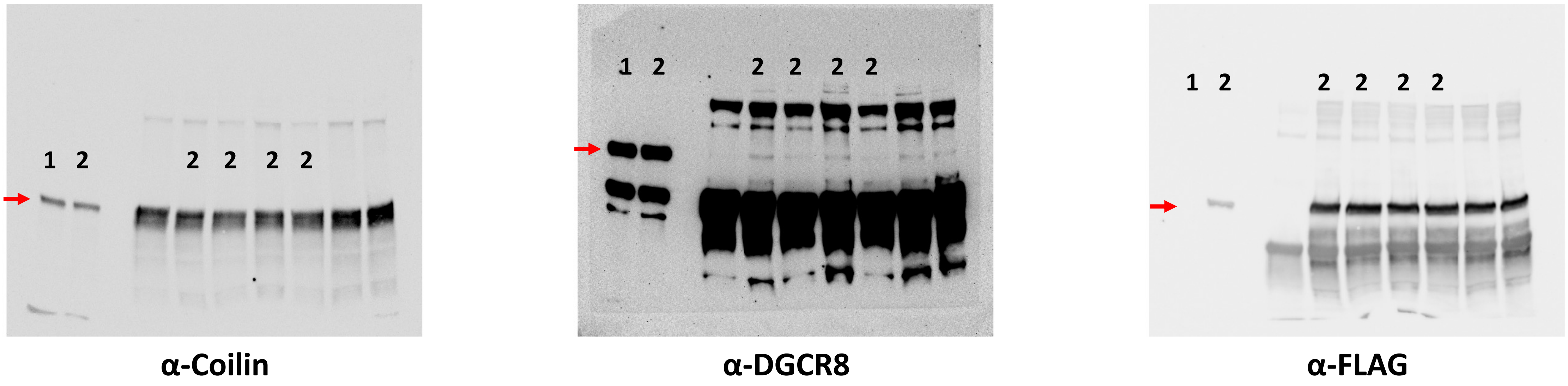

1 – Untransfected
2 – FLAG-METTL3 transfected

Biology Open • Supplementary information

Figure 3C

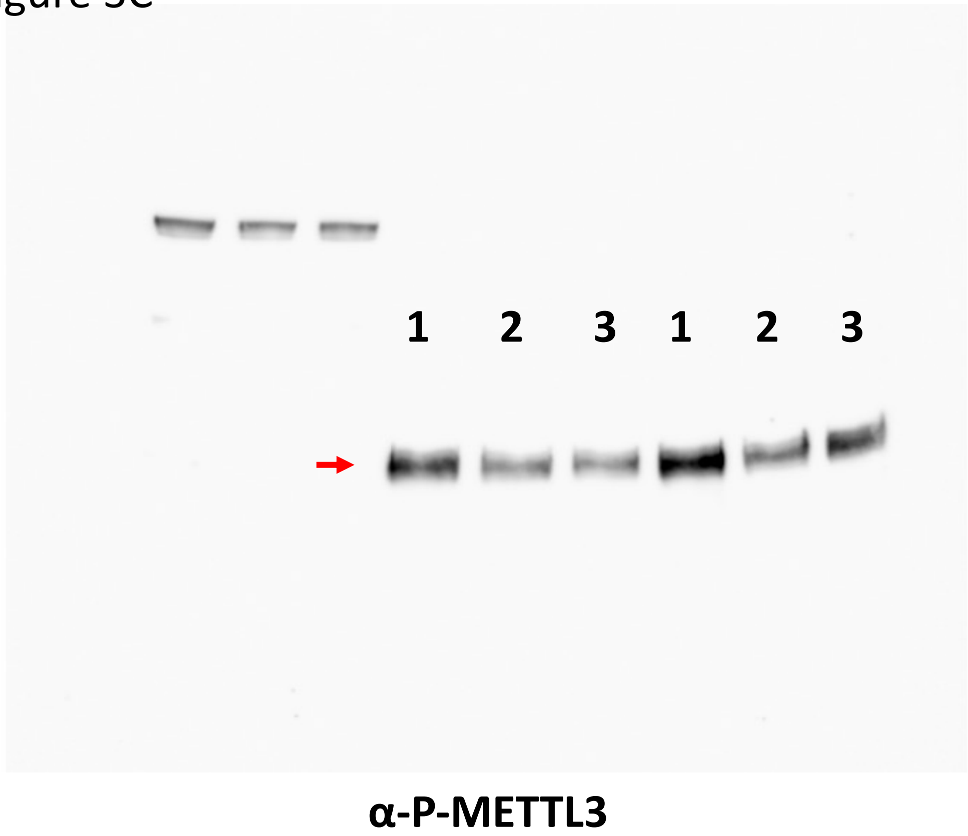

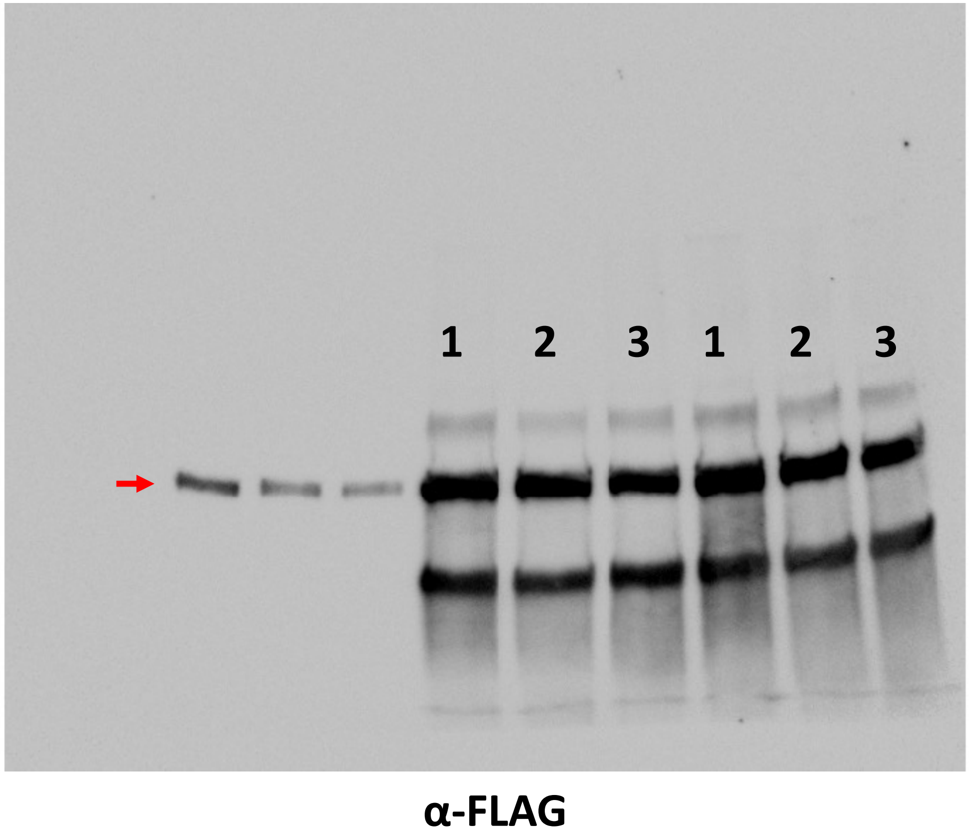

1 – Control KD
2 – Coilin KD
3 – WRAP53 KD

Biology Open • Supplementary information

Figure 4A

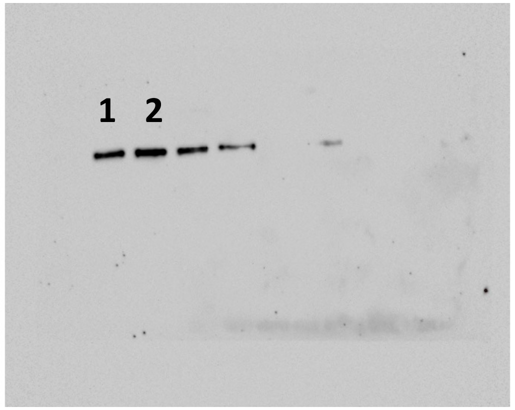

α-METTL3 Cyto Fraction

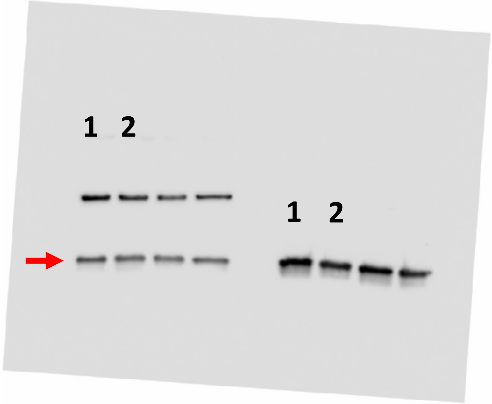

α-U2B'' Cyto/Nuc Fraction

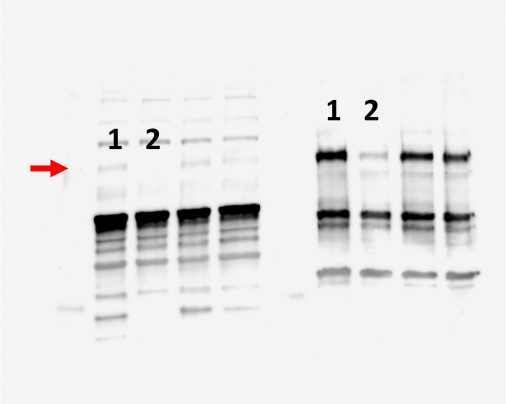

α-Coilin Cyto/Nuc Fraction

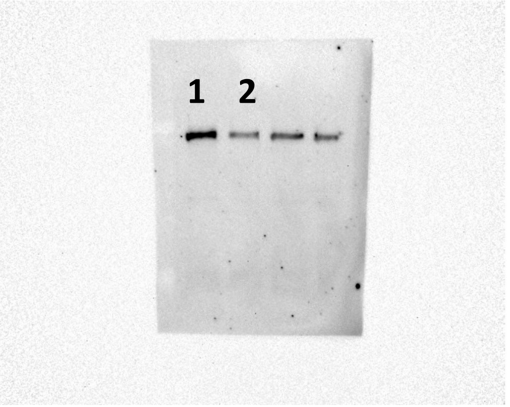

α-METTL3 Nuc Fraction

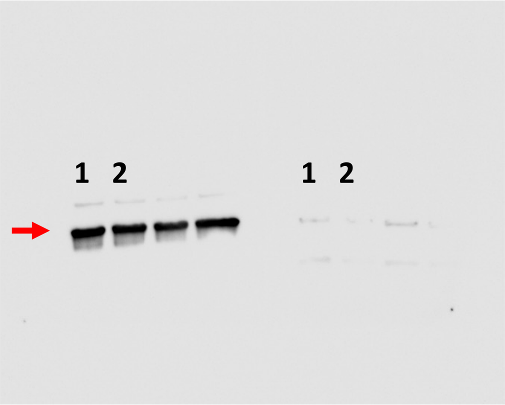

α-GAPDH Cyto/Nuc Fraction

1 – Control KD
2 – Coilin KD

Biology Open • Supplementary information

Figure 4C

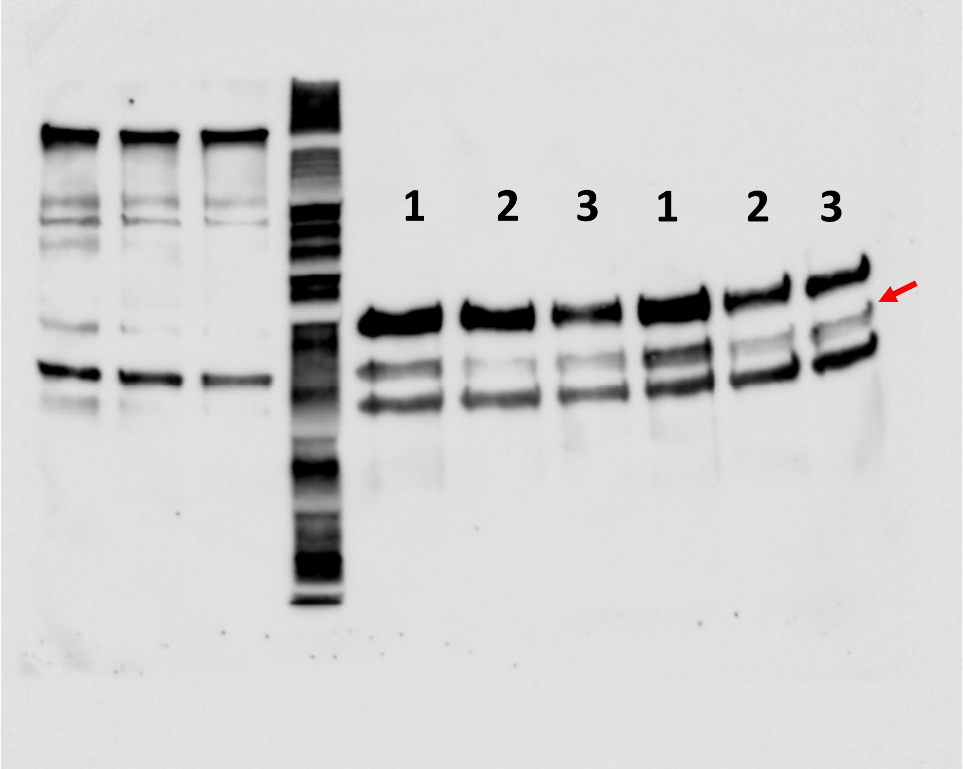

α-METTL14

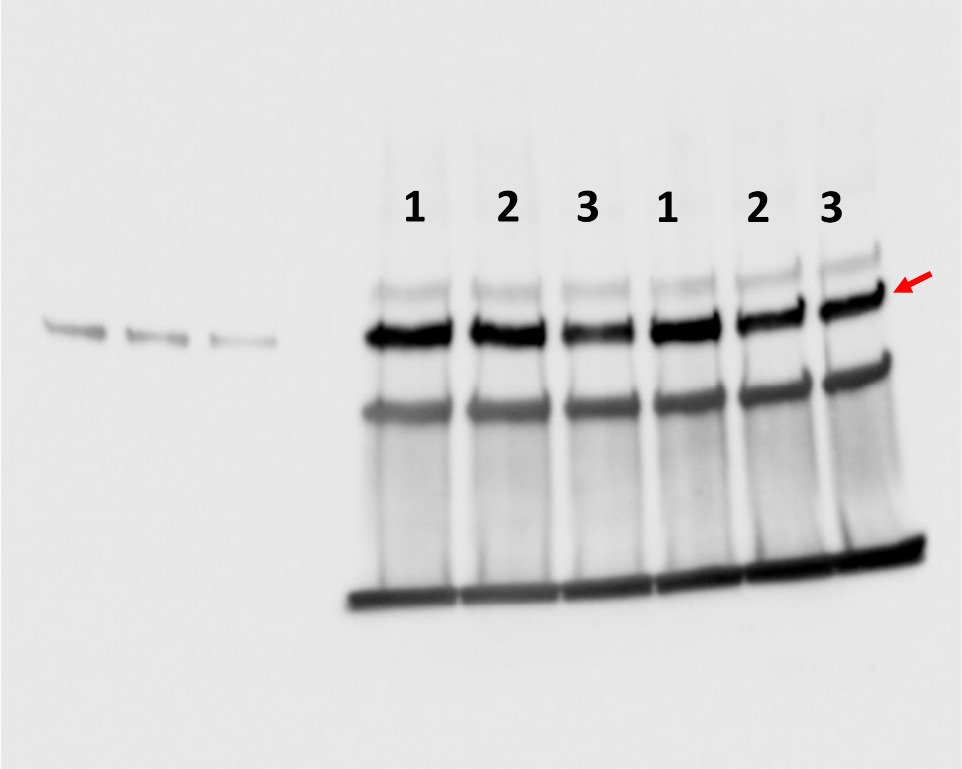

α-FLAG

1 – Control KD
2 – Coilin KD
3 – WRAP53 KD

Biology Open • Supplementary information

Figure 4E

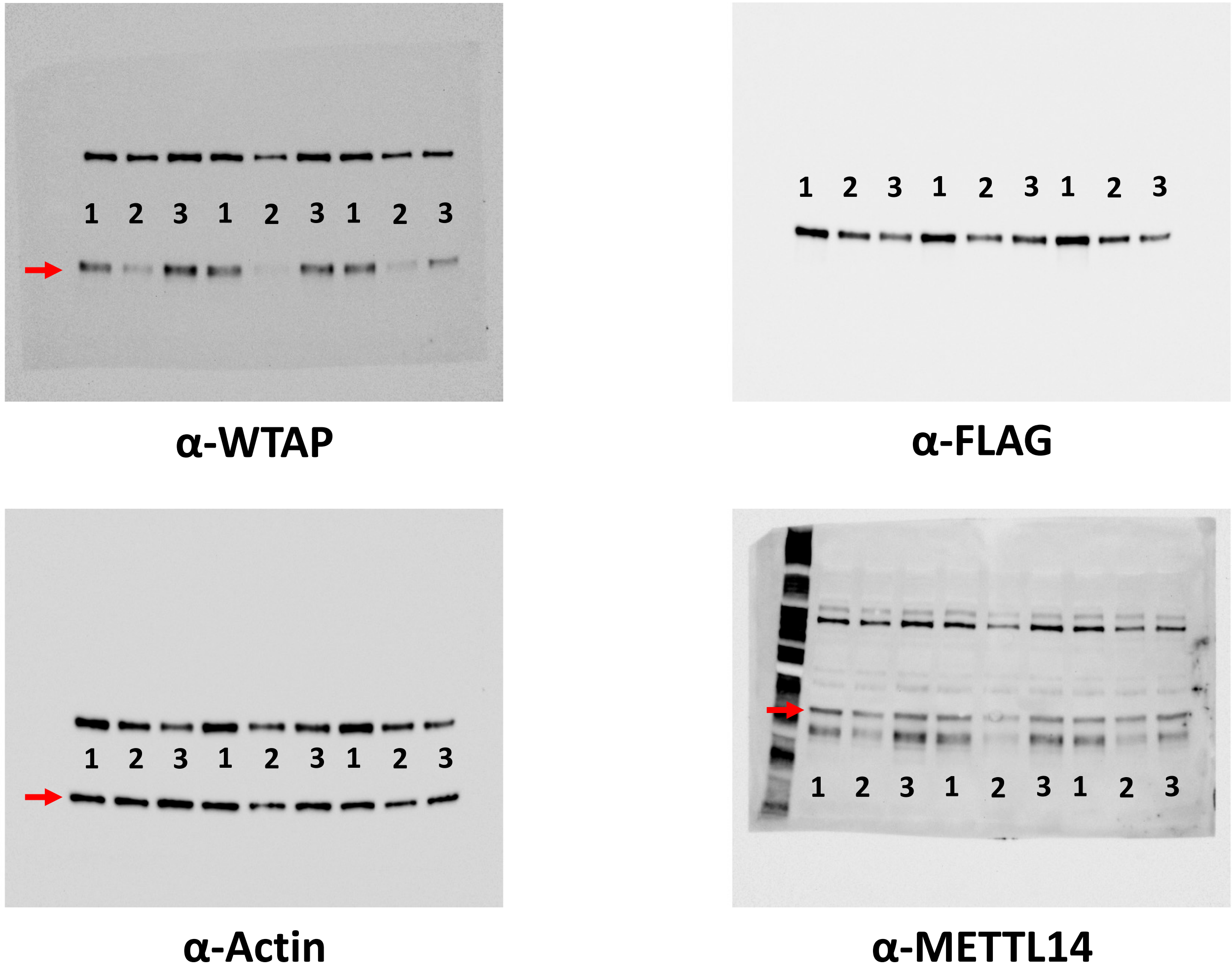

1 – Control KD
2 – Coilin KD
3 – WRAP53 KD

Biology Open • Supplementary information

Figure S3A

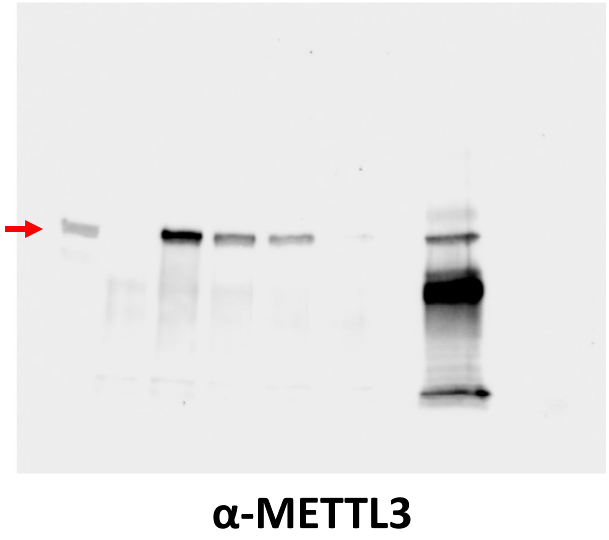

Biology Open • Supplementary information

Figure S3B

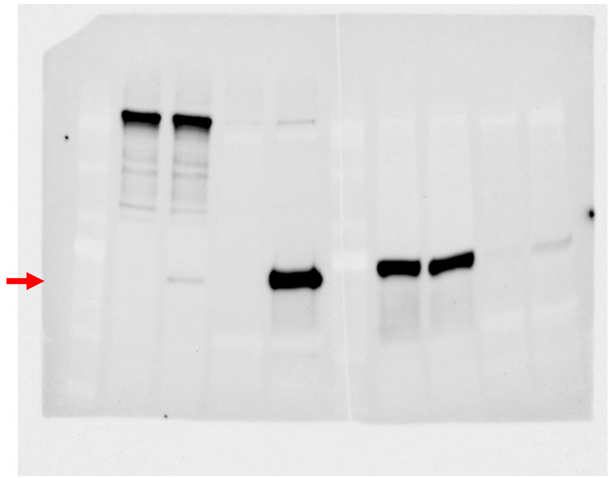

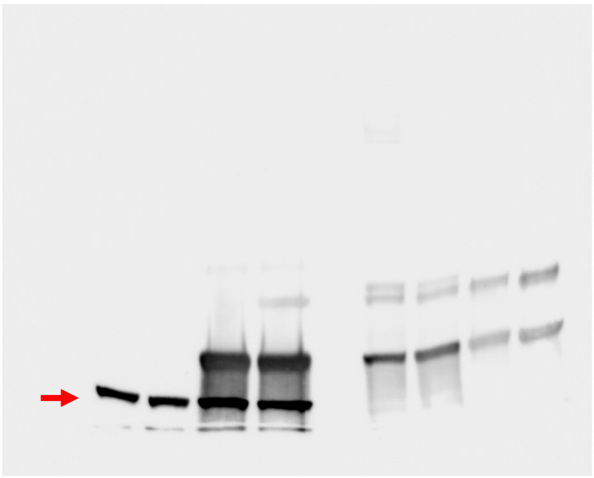

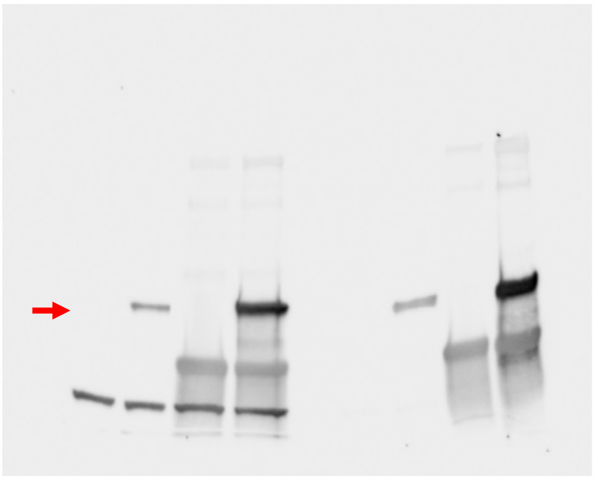

Biology Open • Supplementary information

Figure S4

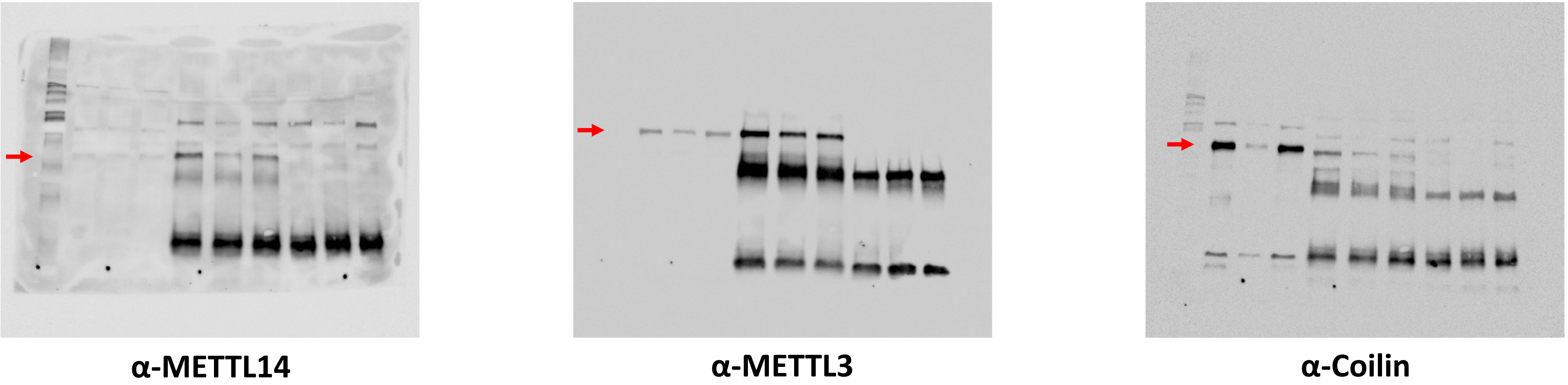

Fig. S5. Blot Transparency.

Table S1. Raw PCR data

Available for download at
https://journals.biologists.com/bio/article-lookup/doi/10.1242/bio.060116#supplementary-data

Biology Open • Supplementary information
